# Supplementary material for: The imprint of star formation on stellar pulsations
Source: Nat Commun. 2022 Sep 19;13:5355. doi: 10.1038/s41467-022-32882-0 (PMC9485136; doi:10.1038/s41467-022-32882-0)
Supplement: Supplementary file 1 — Supplementary Information [file 41467_2022_32882_MOESM1_ESM.pdf]

Supplementary Information:

The imprint of star formation on stellar pulsations

Thomas Steindl et. al

Supplementary figure - Evolutionary tracks in the Kiel diagram

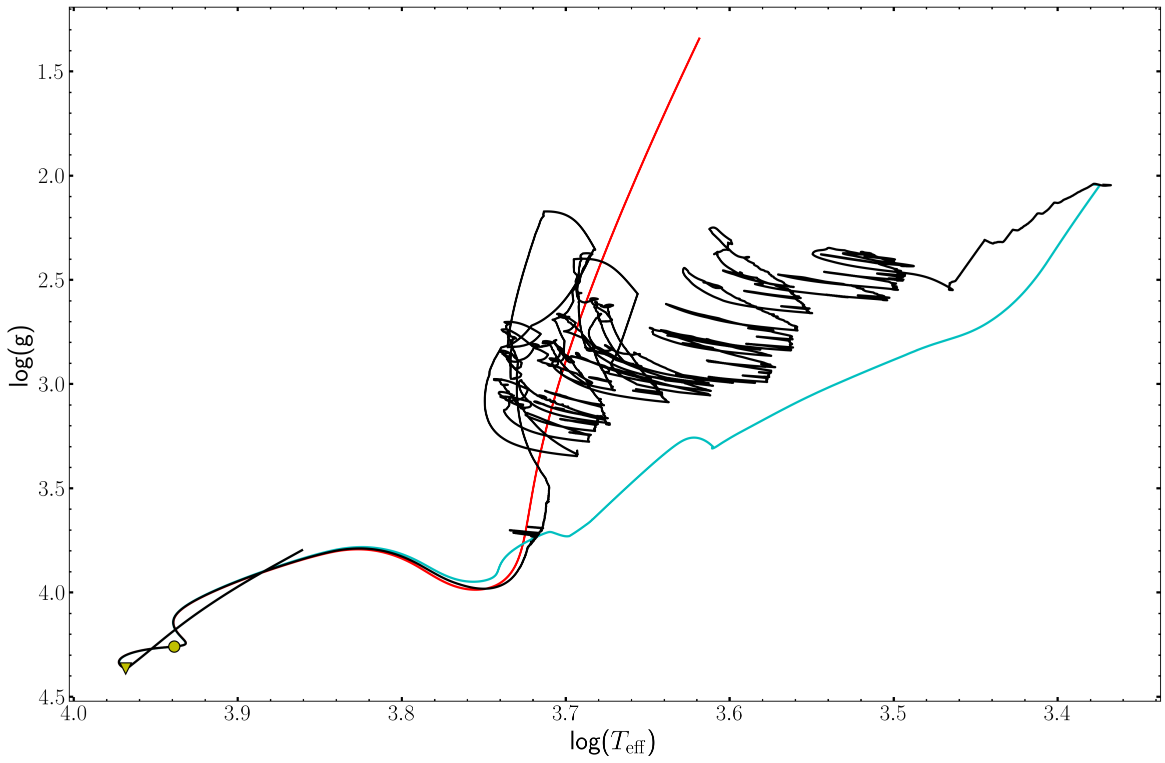

Supplementary Figure 1: Comparison between classical, constant accreting, and a selected disk-mediated model in the Kiel diagram. The red line shows the classical model, the turquoise line shows the constant accretion model and the black line shows the disk-mediated accretion model. The yellow circle marks the predefined pre-main sequence (i.e. central carbon mass fraction drops to a value of 10^{-4}) while the yellow triangle marks the ZAMS (i.e. central hydrogen mass fraction has dropped by 0.01 in comparison to the initial value). All evolutionary tracks shown here have been calculated with the standard input physics.

Supplementary figure - Mass accretion rates for the disk-mediated evolution models

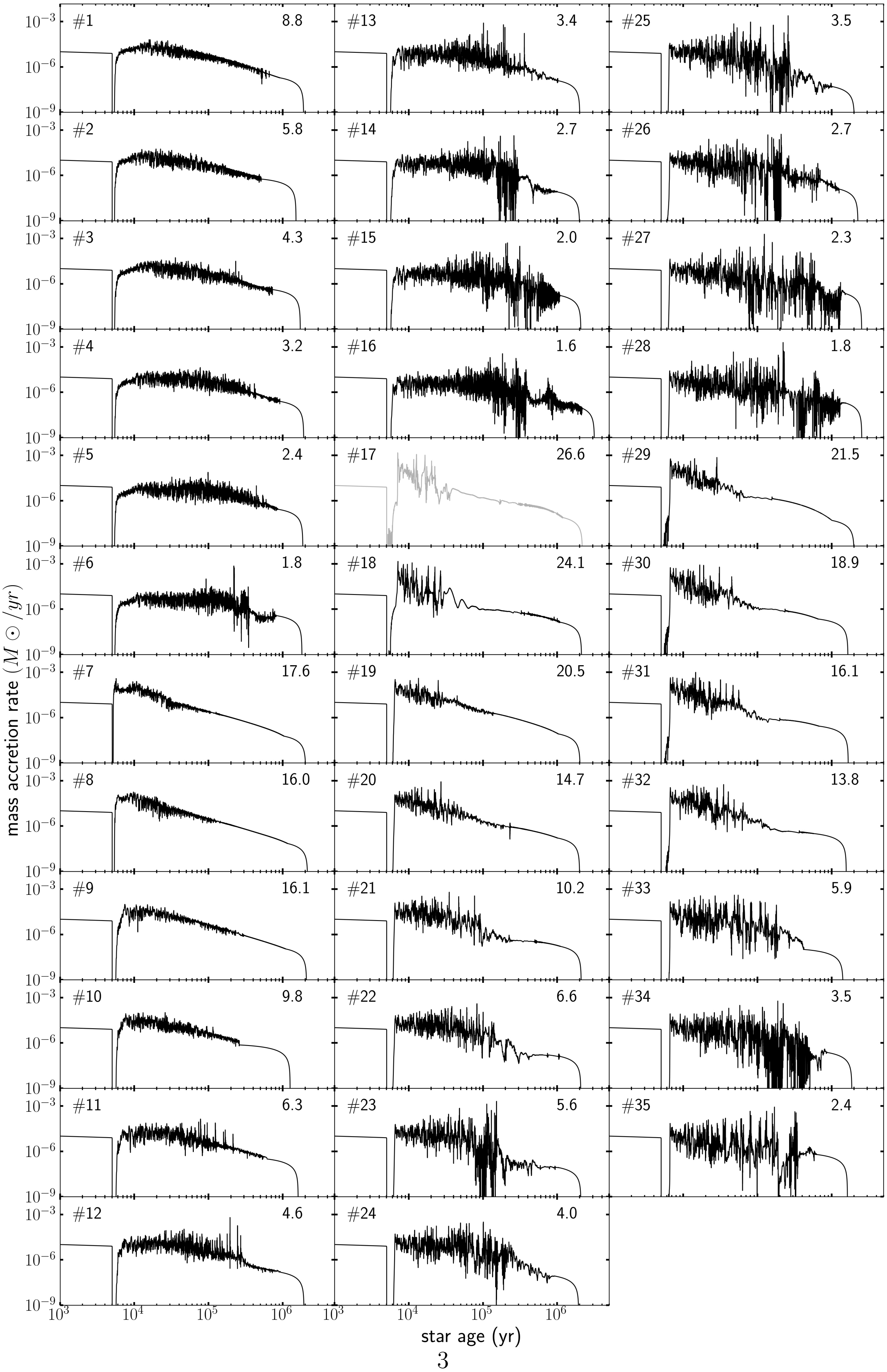

Supplementary Figure 2: Scaled mass accretion rate histories for the stellar evolution calculations with MESA. Each panel shows the mass accretion rate used for the corresponding disk-mediated accretion model. One accretion rate history (#17) does not converge due to the high scaling factor and is omitted from this study. The number in the top right corner of each plot gives the scaling factor rounded to one decimal point.

Supplementary figures - Evolutionary track, internal structure, and frequency differences for the disk-mediated accretion models

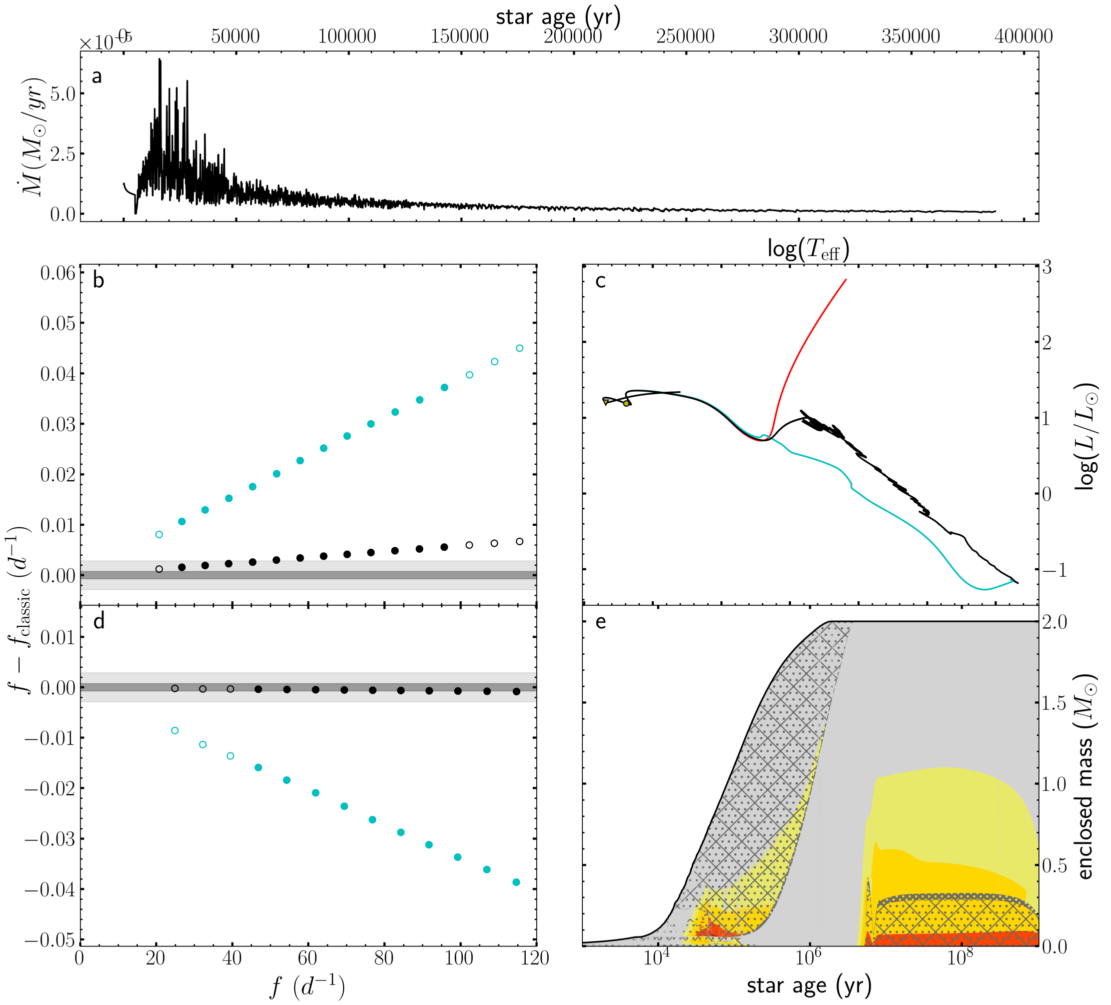

Supplementary Figure 3: Comparison between classical, constant accretion, and disk-mediated accretion model #1 for the standard input physics. **a** The adopted accretion rate as a function of star age. **b** The frequency difference of $l = 1, m = 1$ modes as a function of the pulsation frequency for the predefined pre-main sequence stage. The black (turquoise) circles correspond to differences between the disk-mediated accretion (constant accretion) model and the classical model. Unstable modes are filled, while stable modes are depicted as open circles. The grey areas mark the Rayleigh limit corresponding to 4-year Kepler (dark grey) and 357-days TESS light curves (light grey). **c** The evolution of the classical model (red), the constant accretion model (turquoise) and

the disk-mediated accretion model (black) in the Hertzsprung-Russell. **d** Same as panel b but for the evolution model at the ZAMS. **e** Kippenhahn diagram of the evolution (same style as in Figure 1).

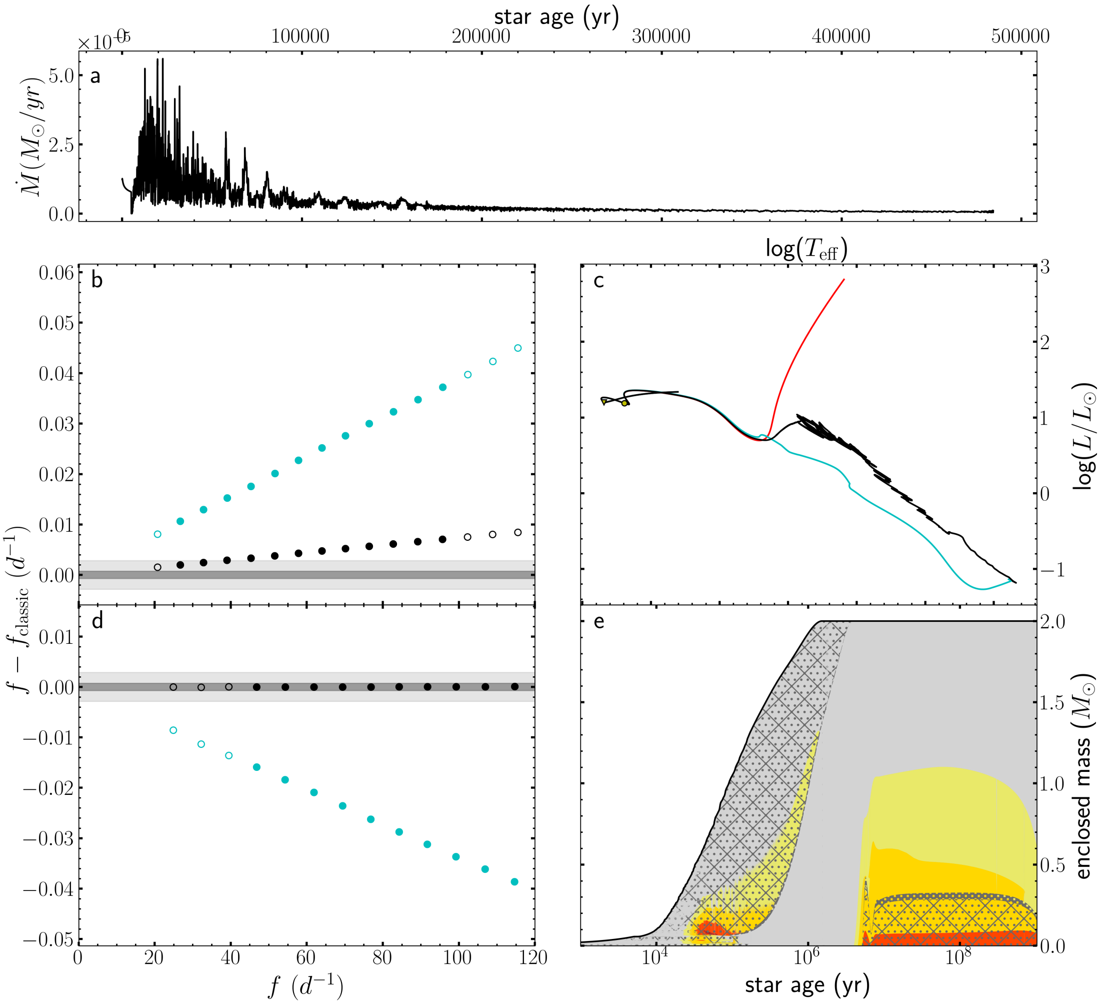

Supplementary Figure 4: Comparison between classical, constant accretion, and disk-mediated accretion model #2 for the standard input physics. a The adopted accretion rate as a function of star age. **b** The frequency difference of $l = 1, m = 1$ modes as a function of the pulsation frequency for the predefined pre-main sequence stage. The black (turquoise) circles correspond to differences between the disk-mediated accretion (constant accretion) model and the classical model. Unstable modes are filled, while stable modes are depicted as open circles. The grey areas mark the Rayleigh limit corresponding to 4-year Kepler (dark grey) and 357-days TESS light curves (light grey). **c** The evolution of the classical model (red), the constant accretion model (turquoise) and the disk-mediated accretion model (black) in the Hertzsprung-Russell. **d** Same as panel b but for the evolution model at the ZAMS. **e** Kippenhahn diagram of the evolution (same style as in Figure 1).

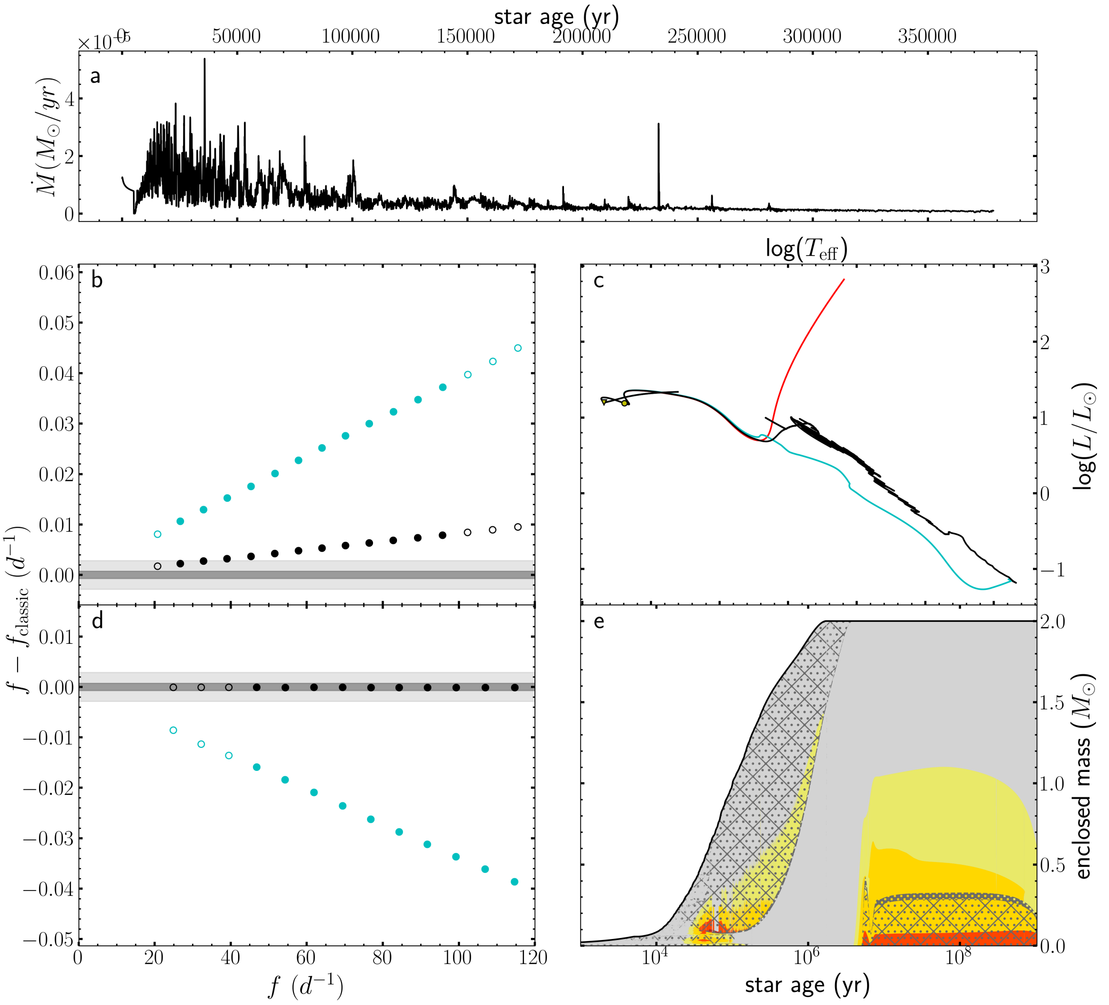

Supplementary Figure 5: Comparison between classical, constant accretion, and disk-mediated accretion model #3 for the standard input physics. **a** The adopted accretion rate as a function of star age. **b** The frequency difference of $l = 1, m = 1$ modes as a function of the pulsation frequency for the predefined pre-main sequence stage. The black (turquoise) circles correspond to differences between the disk-mediated accretion (constant accretion) model and the classical model. Unstable modes are filled, while stable modes are depicted as open circles. The grey areas mark the Rayleigh limit corresponding to 4-year Kepler (dark grey) and 357-days TESS light curves (light grey). **c** The evolution of the classical model (red), the constant accretion model (turquoise) and the disk-mediated accretion model (black) in the Hertzsprung-Russell. **d** Same as panel b but for the evolution model at the ZAMS. **e** Kippenhahn diagram of the evolution (same style as in Figure 1).

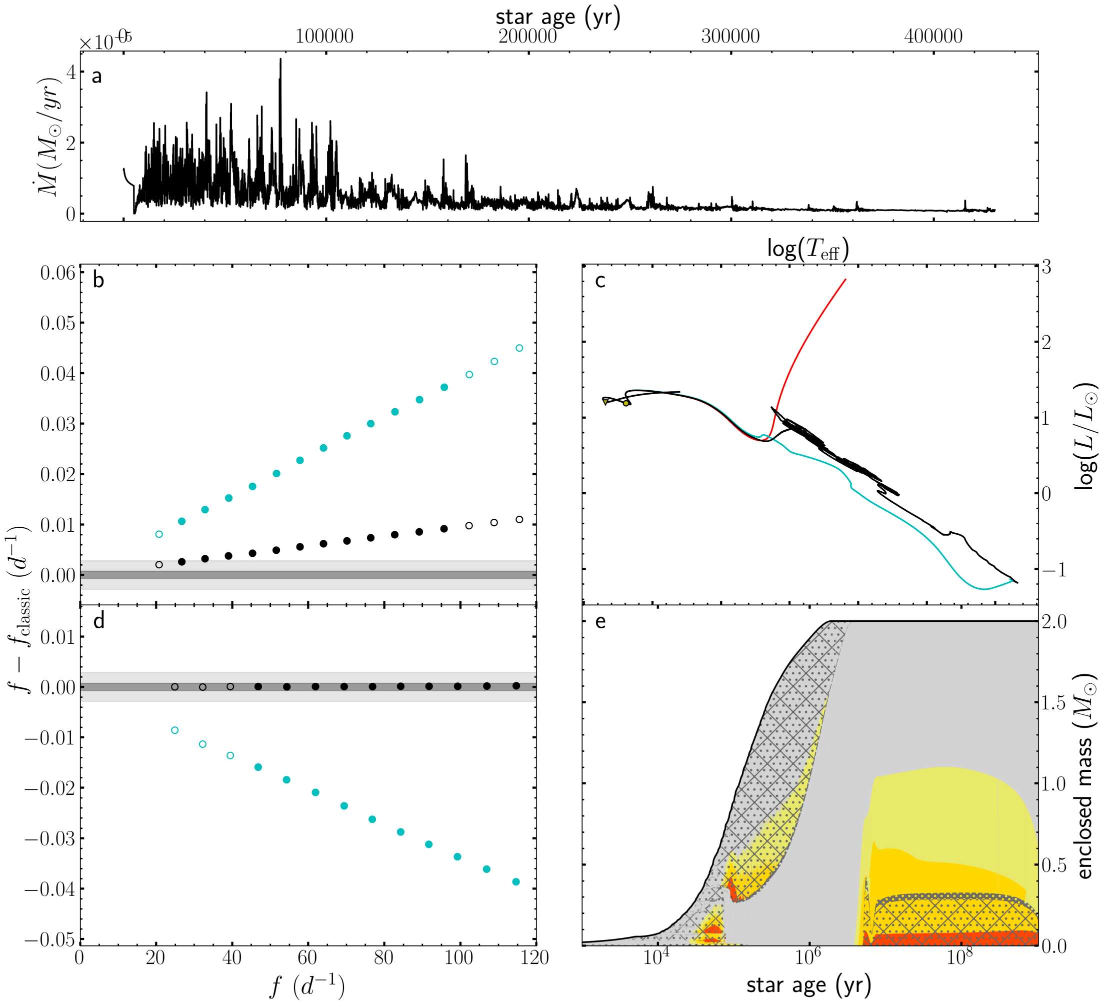

Supplementary Figure 6: Comparison between classical, constant accretion, and disk-mediated accretion model #4 for the standard input physics. a The adopted accretion rate as a function of star age. **b** The frequency difference of $l = 1, m = 1$ modes as a function of the pulsation frequency for the predefined pre-main sequence stage. The black (turquoise) circles correspond to differences between the disk-mediated accretion (constant accretion) model and the classical model. Unstable modes are filled, while stable modes are depicted as open circles. The grey areas mark the Rayleigh limit corresponding to 4-year Kepler (dark grey) and 357-days TESS light curves (light grey). **c** The evolution of the classical model (red), the constant accretion model (turquoise) and the disk-mediated accretion model (black) in the Hertzsprung-Russell. **d** Same as panel b but for the evolution model at the ZAMS. **e** Kippenhahn diagram of the evolution (same style as in Figure 1).

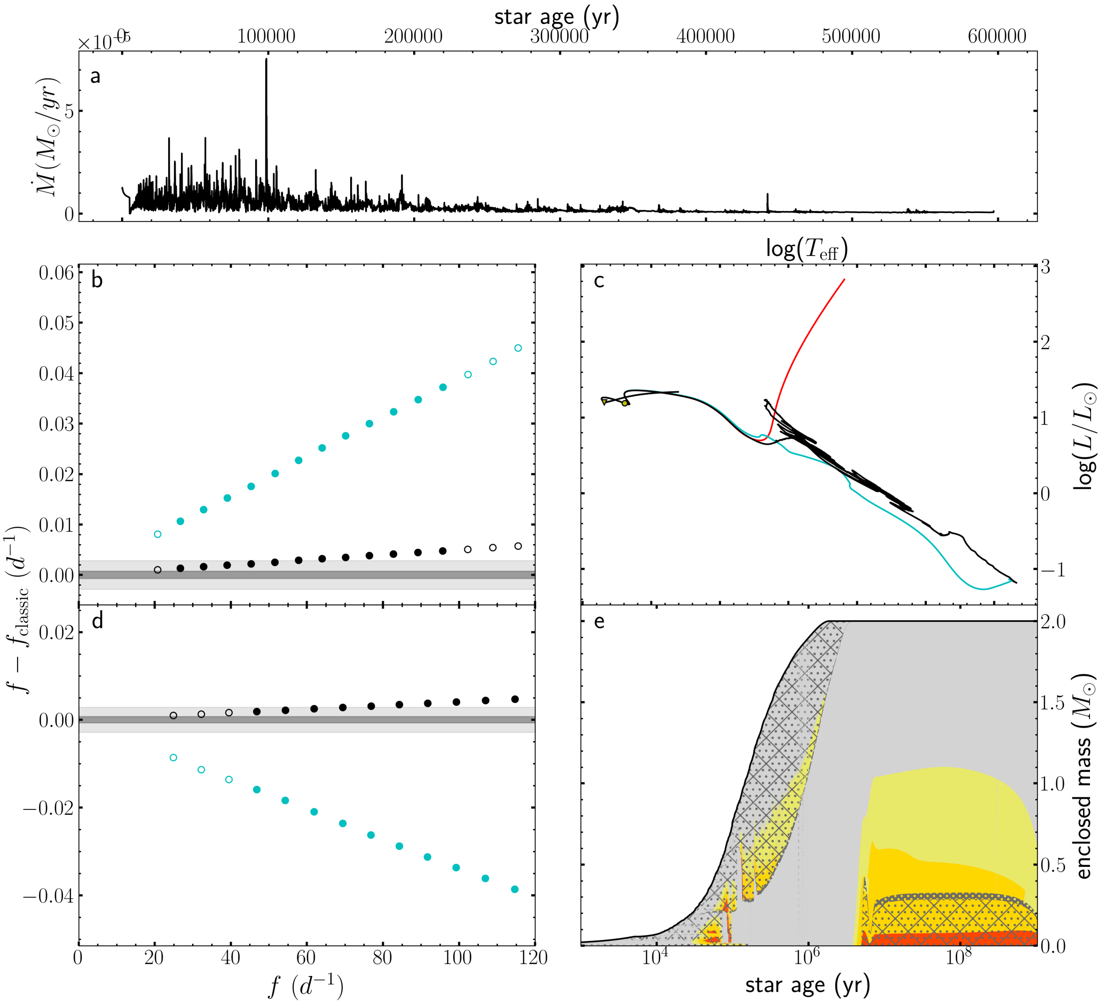

Supplementary Figure 7: Comparison between classical, constant accretion, and disk-mediated accretion model #5 for the standard input physics. a The adopted accretion rate as a function of star age. **b** The frequency difference of $l = 1, m = 1$ modes as a function of the pulsation frequency for the predefined pre-main sequence stage. The black (turquoise) circles correspond to differences between the disk-mediated accretion (constant accretion) model and the classical model. Unstable modes are filled, while stable modes are depicted as open circles. The grey areas mark the Rayleigh limit corresponding to 4-year Kepler (dark grey) and 357-days TESS light curves (light grey). **c** The evolution of the classical model (red), the constant accretion model (turquoise) and the disk-mediated accretion model (black) in the Hertzsprung-Russell. **d** Same as panel b but for the evolution model at the ZAMS. **e** Kippenhahn diagram of the evolution (same style as in Figure 1).

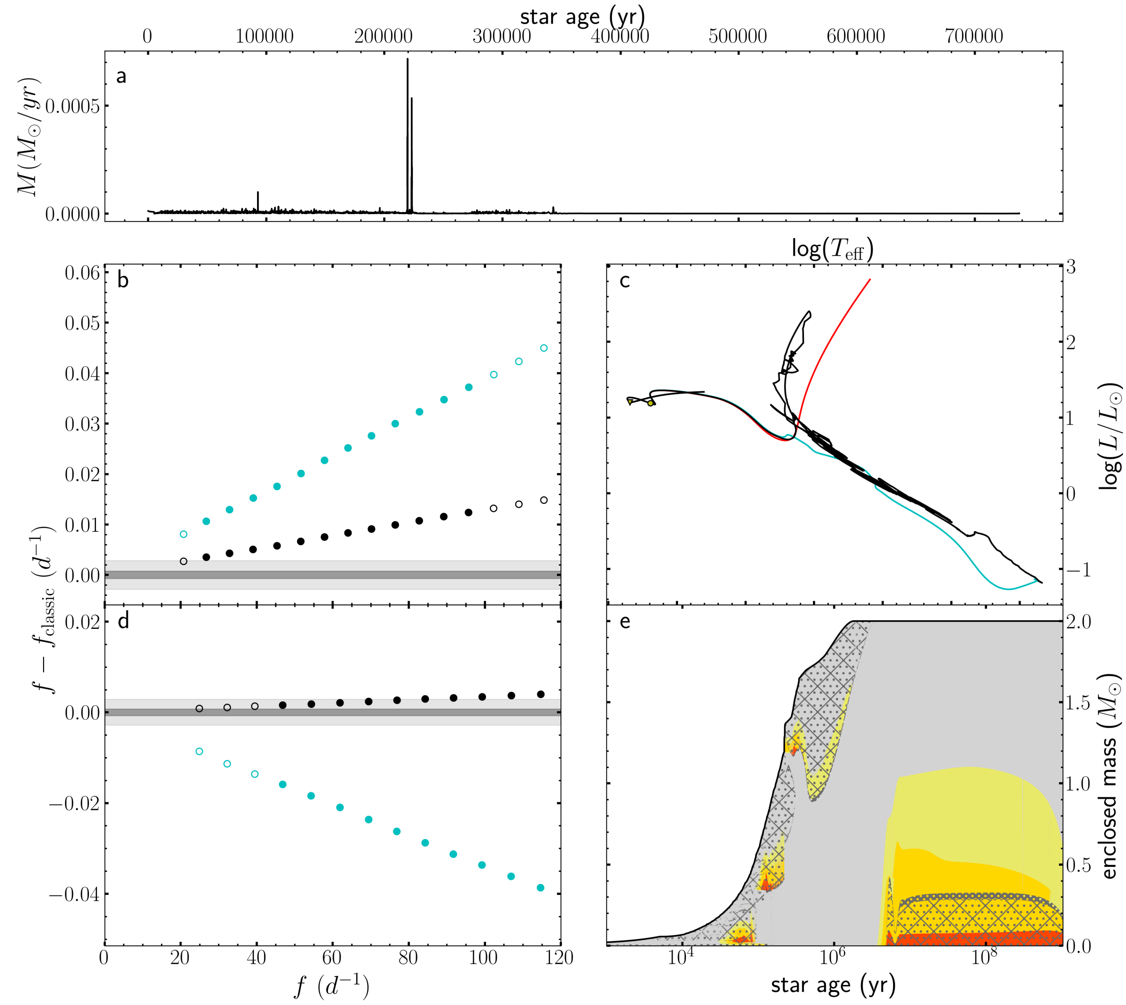

Supplementary Figure 8: Comparison between classical, constant accretion, and disk-mediated accretion model #6 for the standard input physics. **a** The adopted accretion rate as a function of star age. **b** The frequency difference of $l = 1, m = 1$ modes as a function of the pulsation frequency for the predefined pre-main sequence stage. The black (turquoise) circles correspond to differences between the disk-mediated accretion (constant accretion) model and the classical model. Unstable modes are filled, while stable modes are depicted as open circles. The grey areas mark the Rayleigh limit corresponding to 4-year Kepler (dark grey) and 357-days TESS light curves (light grey). **c** The evolution of the classical model (red), the constant accretion model (turquoise) and the disk-mediated accretion model (black) in the Hertzsprung-Russell. **d** Same as panel b but for the evolution model at the ZAMS. **e** Kippenhahn diagram of the evolution (same style as in Figure 1).

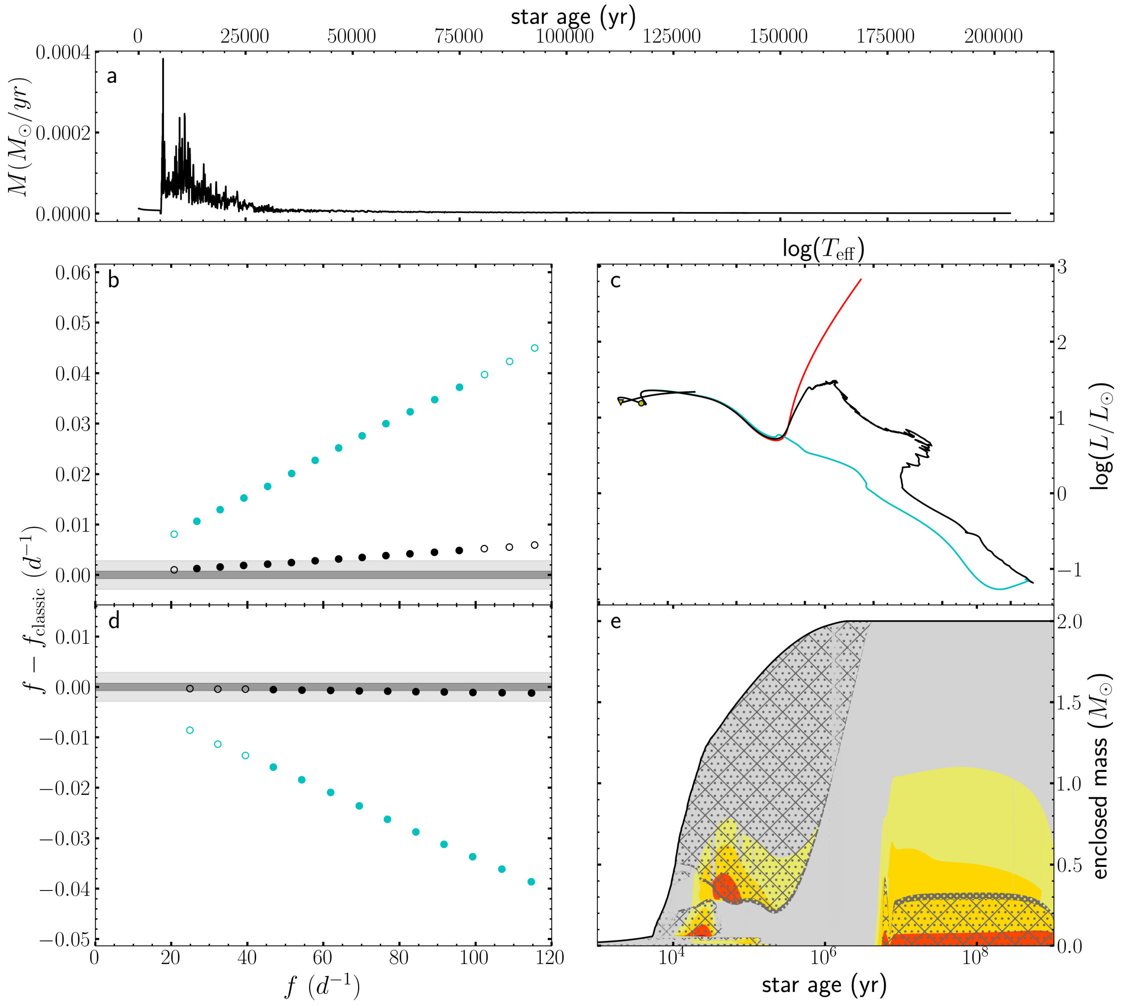

Supplementary Figure 9: Comparison between classical, constant accretion, and disk-mediated accretion model #7 for the standard input physics. a The adopted accretion rate as a function of star age. **b** The frequency difference of $l = 1, m = 1$ modes as a function of the pulsation frequency for the predefined pre-main sequence stage. The black (turquoise) circles correspond to differences between the disk-mediated accretion (constant accretion) model and the classical model. Unstable modes are filled, while stable modes are depicted as open circles. The grey areas mark the Rayleigh limit corresponding to 4-year Kepler (dark grey) and 357-days TESS light curves (light grey). **c** The evolution of the classical model (red), the constant accretion model (turquoise) and the disk-mediated accretion model (black) in the Hertzsprung-Russell. **d** Same as panel b but for the evolution model at the ZAMS. **e** Kippenhahn diagram of the evolution (same style as in Figure 1).

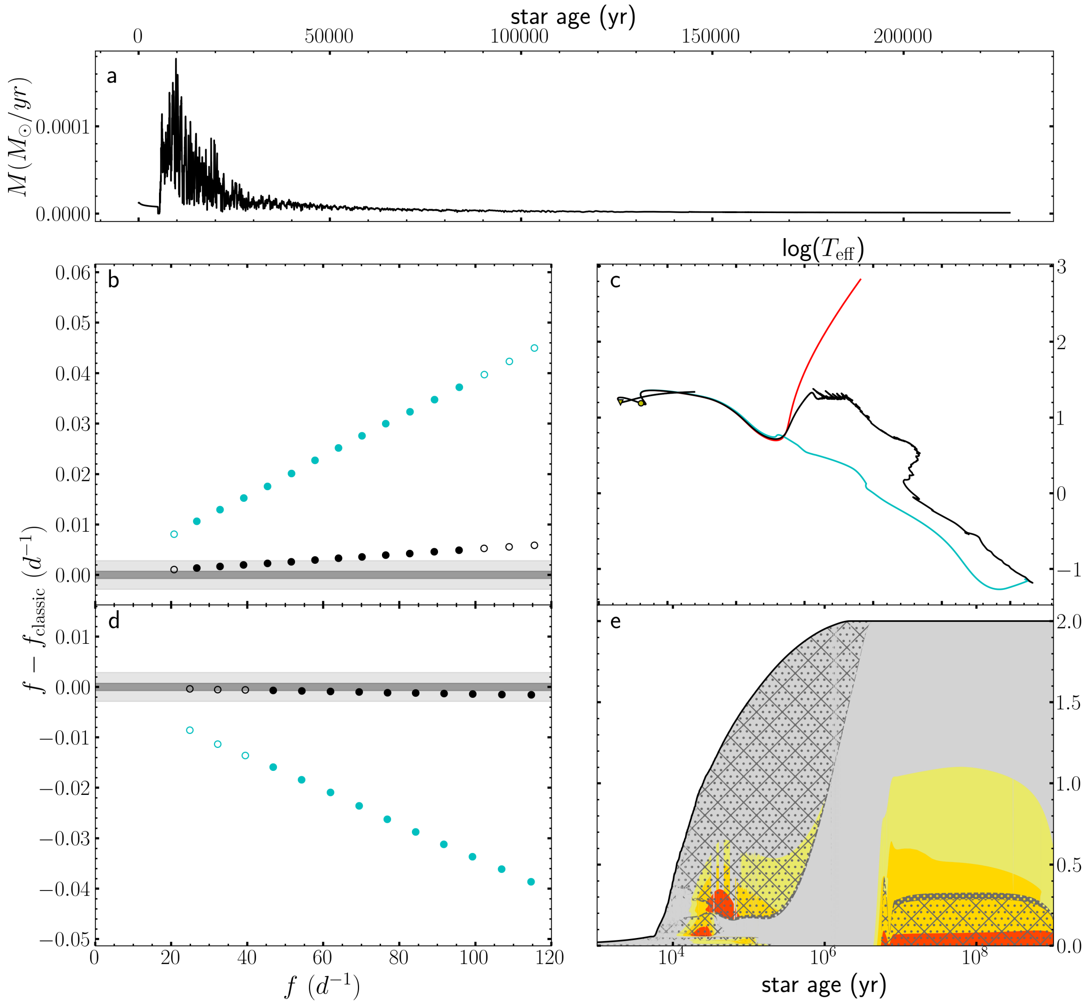

Supplementary Figure 10: Comparison between classical, constant accretion, and disk-mediated accretion model #8 for the standard input physics. **a** The adopted accretion rate as a function of star age. **b** The frequency difference of $l = 1, m = 1$ modes as a function of the pulsation frequency for the predefined pre-main sequence stage. The black (turquoise) circles correspond to differences between the disk-mediated accretion (constant accretion) model and the classical model. Unstable modes are filled, while stable modes are depicted as open circles. The grey areas mark the Rayleigh limit corresponding to 4-year Kepler (dark grey) and 357-days TESS light curves (light grey). **c** The evolution of the classical model (red), the constant accretion model (turquoise) and the disk-mediated accretion model (black) in the Hertzsprung-Russell. **d** Same as panel b but for the evolution model at the ZAMS. **e** Kippenhahn diagram of the evolution (same style as in Figure 1).

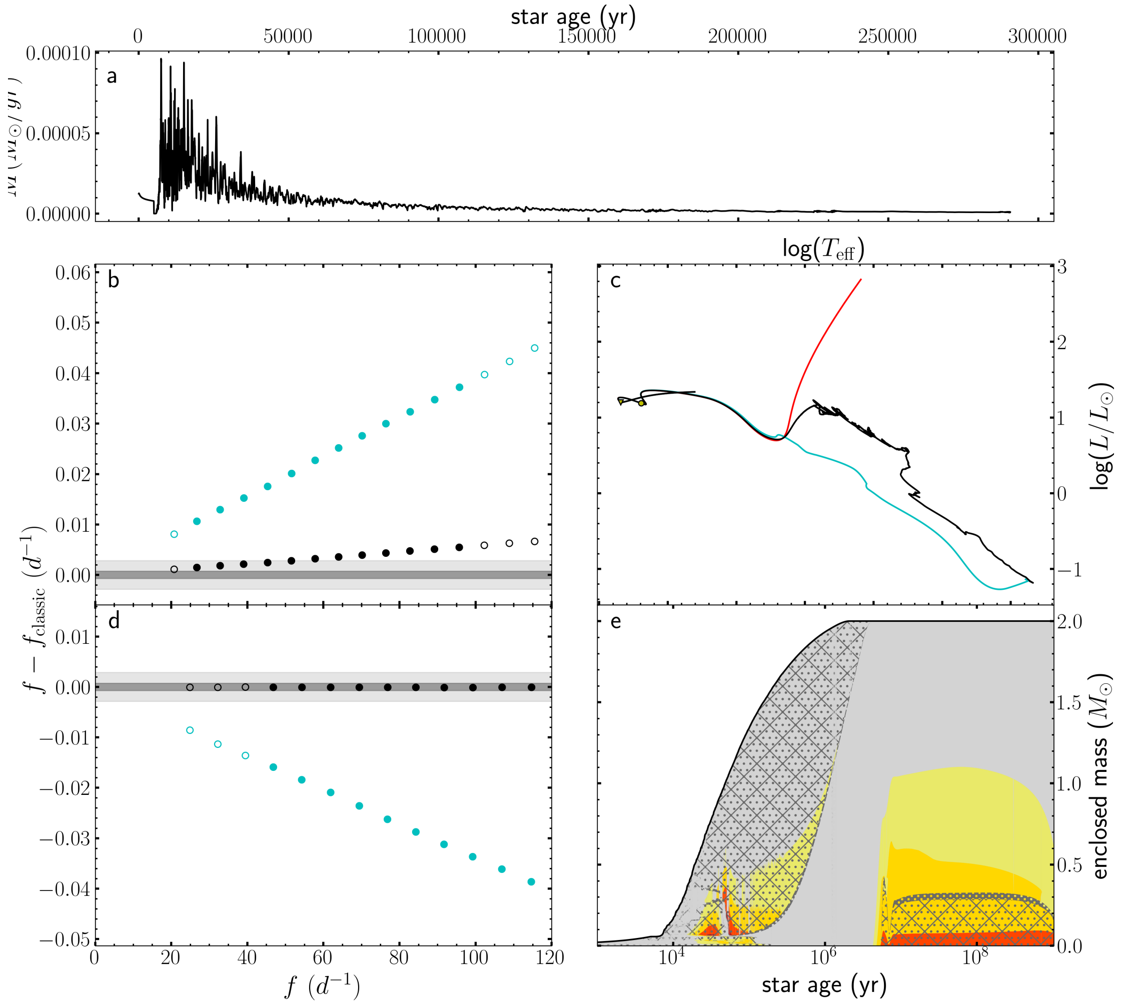

Supplementary Figure 11: Comparison between classical, constant accretion, and disk-mediated accretion model #9 for the standard input physics. **a** The adopted accretion rate as a function of star age. **b** The frequency difference of $l = 1, m = 1$ modes as a function of the pulsation frequency for the predefined pre-main sequence stage. The black (turquoise) circles correspond to differences between the disk-mediated accretion (constant accretion) model and the classical model. Unstable modes are filled, while stable modes are depicted as open circles. The grey areas mark the Rayleigh limit corresponding to 4-year Kepler (dark grey) and 357-days TESS light curves (light grey). **c** The evolution of the classical model (red), the constant accretion model (turquoise) and the disk-mediated accretion model (black) in the Hertzsprung-Russell. **d** Same as panel b but for the evolution model at the ZAMS. **e** Kippenhahn diagram of the evolution (same style as in Figure 1).

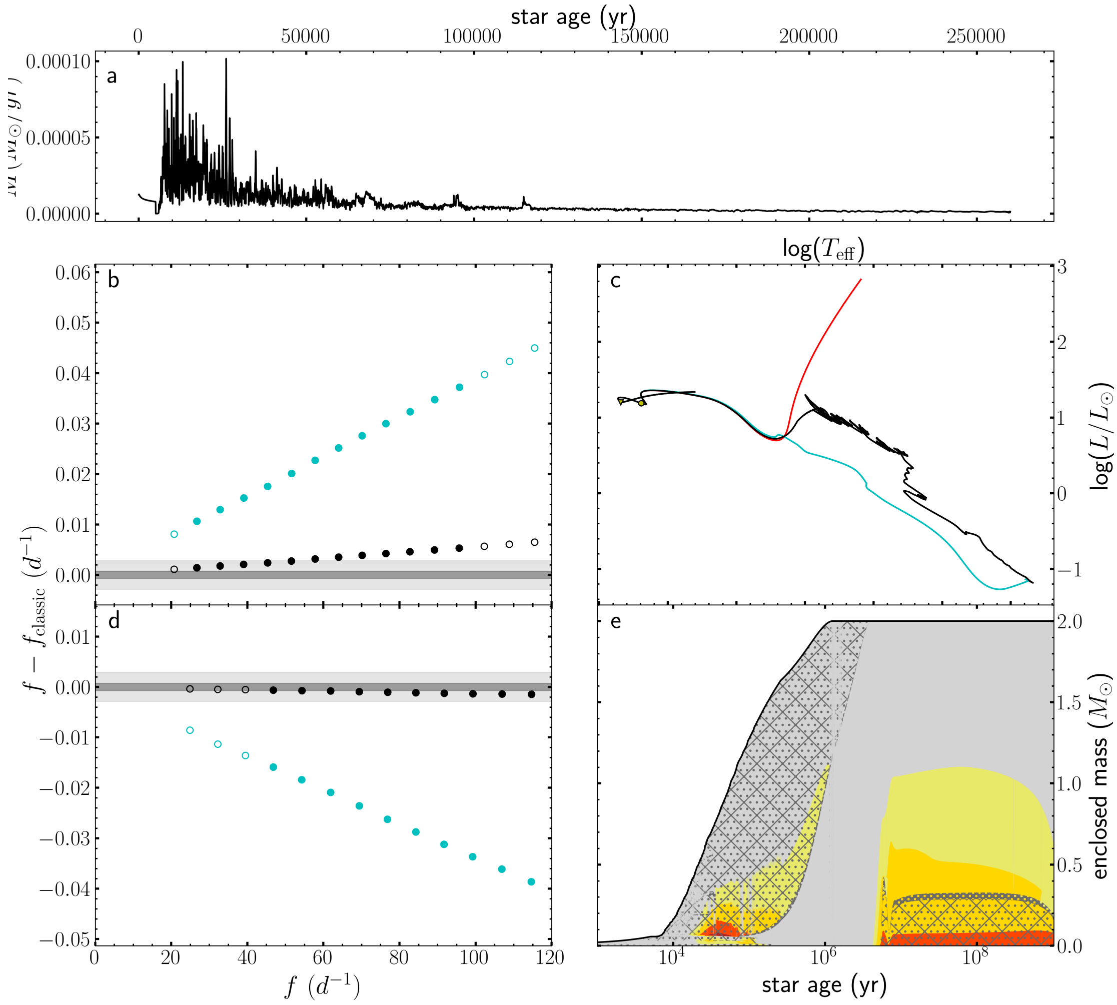

Supplementary Figure 12: Comparison between classical, constant accretion, and disk-mediated accretion model #10 for the standard input physics. a The adopted accretion rate as a function of star age. **b** The frequency difference of $l = 1, m = 1$ modes as a function of the pulsation frequency for the predefined pre-main sequence stage. The black (turquoise) circles correspond to differences between the disk-mediated accretion (constant accretion) model and the classical model. Unstable modes are filled, while stable modes are depicted as open circles. The grey areas mark the Rayleigh limit corresponding to 4-year Kepler (dark grey) and 357-days TESS light curves (light grey). **c** The evolution of the classical model (red), the constant accretion model (turquoise) and the disk-mediated accretion model (black) in the Hertzsprung-Russell. **d** Same as panel b but for the evolution model at the ZAMS. **e** Kippenhahn diagram of the evolution (same style as in Figure 1).

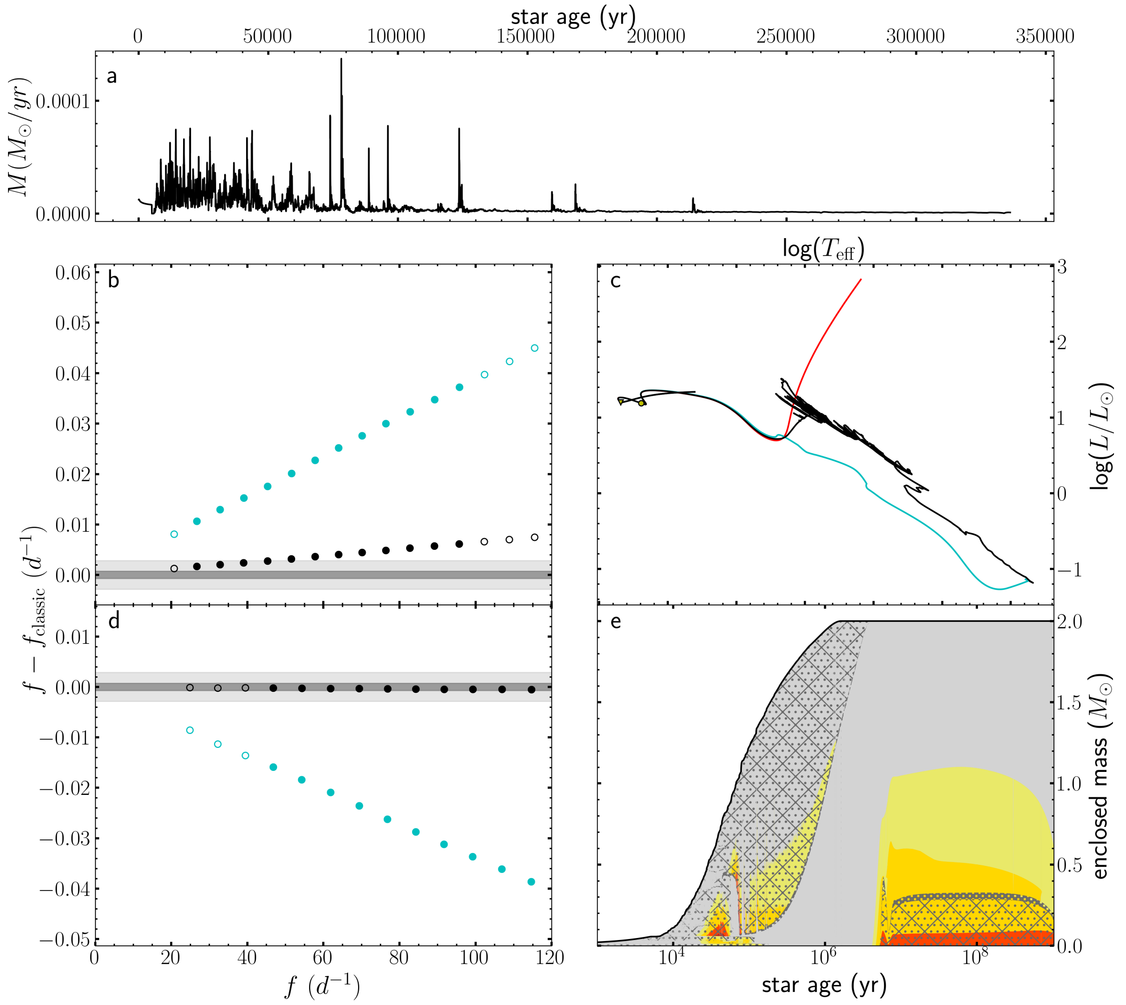

Supplementary Figure 13: Comparison between classical, constant accretion, and disk-mediated accretion model #11 for the standard input physics. a The adopted accretion rate as a function of star age. **b** The frequency difference of $l = 1, m = 1$ modes as a function of the pulsation frequency for the predefined pre-main sequence stage. The black (turquoise) circles correspond to differences between the disk-mediated accretion (constant accretion) model and the classical model. Unstable modes are filled, while stable modes are depicted as open circles. The grey areas mark the Rayleigh limit corresponding to 4-year Kepler (dark grey) and 357-days TESS light curves (light grey). **c** The evolution of the classical model (red), the constant accretion model (turquoise) and the disk-mediated accretion model (black) in the Hertzsprung-Russell. **d** Same as panel b but for the evolution model at the ZAMS. **e** Kippenhahn diagram of the evolution (same style as in Figure 1).

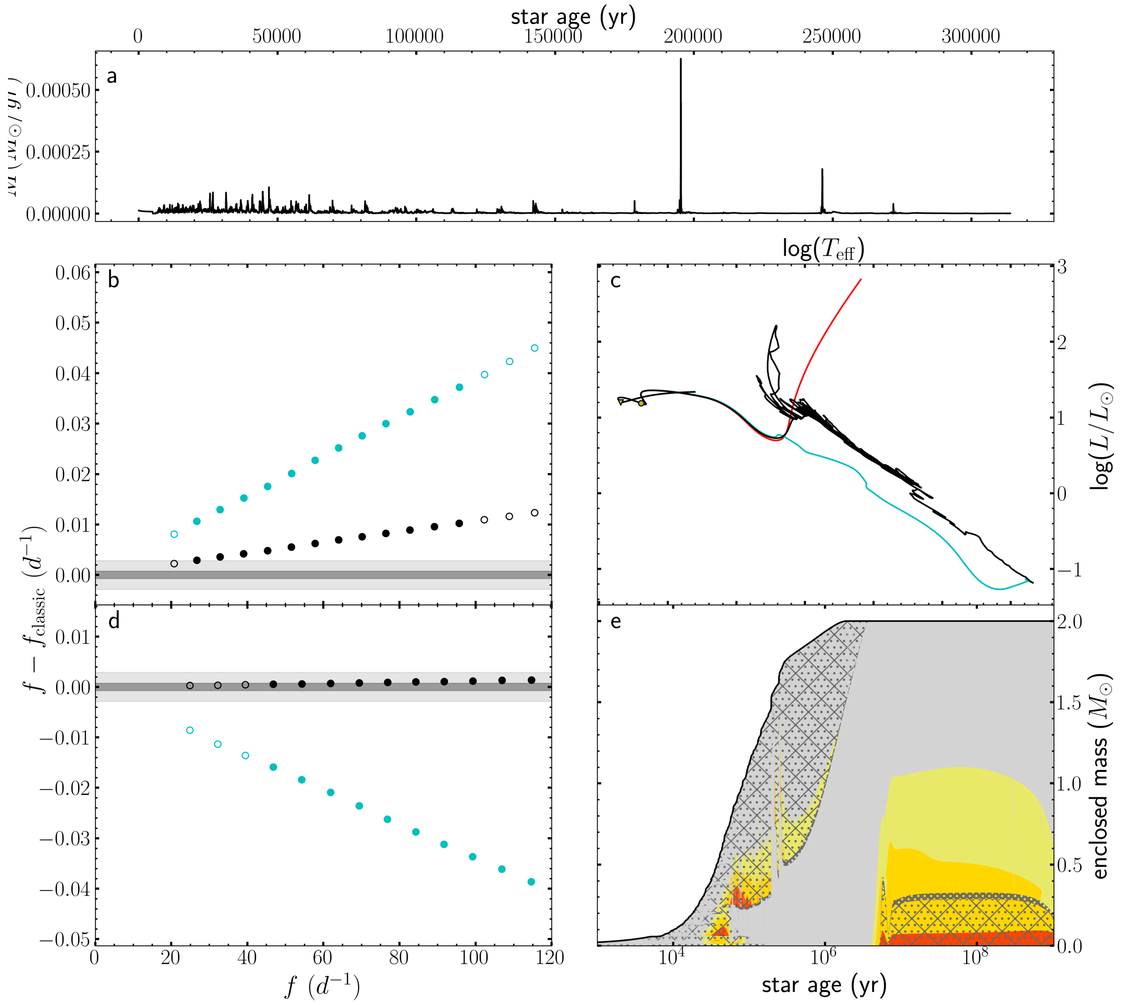

Supplementary Figure 14: Comparison between classical, constant accretion, and disk-mediated accretion model #12 for the standard input physics. a The adopted accretion rate as a function of star age. **b** The frequency difference of $l = 1, m = 1$ modes as a function of the pulsation frequency for the predefined pre-main sequence stage. The black (turquoise) circles correspond to differences between the disk-mediated accretion (constant accretion) model and the classical model. Unstable modes are filled, while stable modes are depicted as open circles. The grey areas mark the Rayleigh limit corresponding to 4-year Kepler (dark grey) and 357-days TESS light curves (light grey). **c** The evolution of the classical model (red), the constant accretion model (turquoise) and the disk-mediated accretion model (black) in the Hertzsprung-Russell. **d** Same as panel b but for the evolution model at the ZAMS. **e** Kippenhahn diagram of the evolution (same style as in Figure 1).

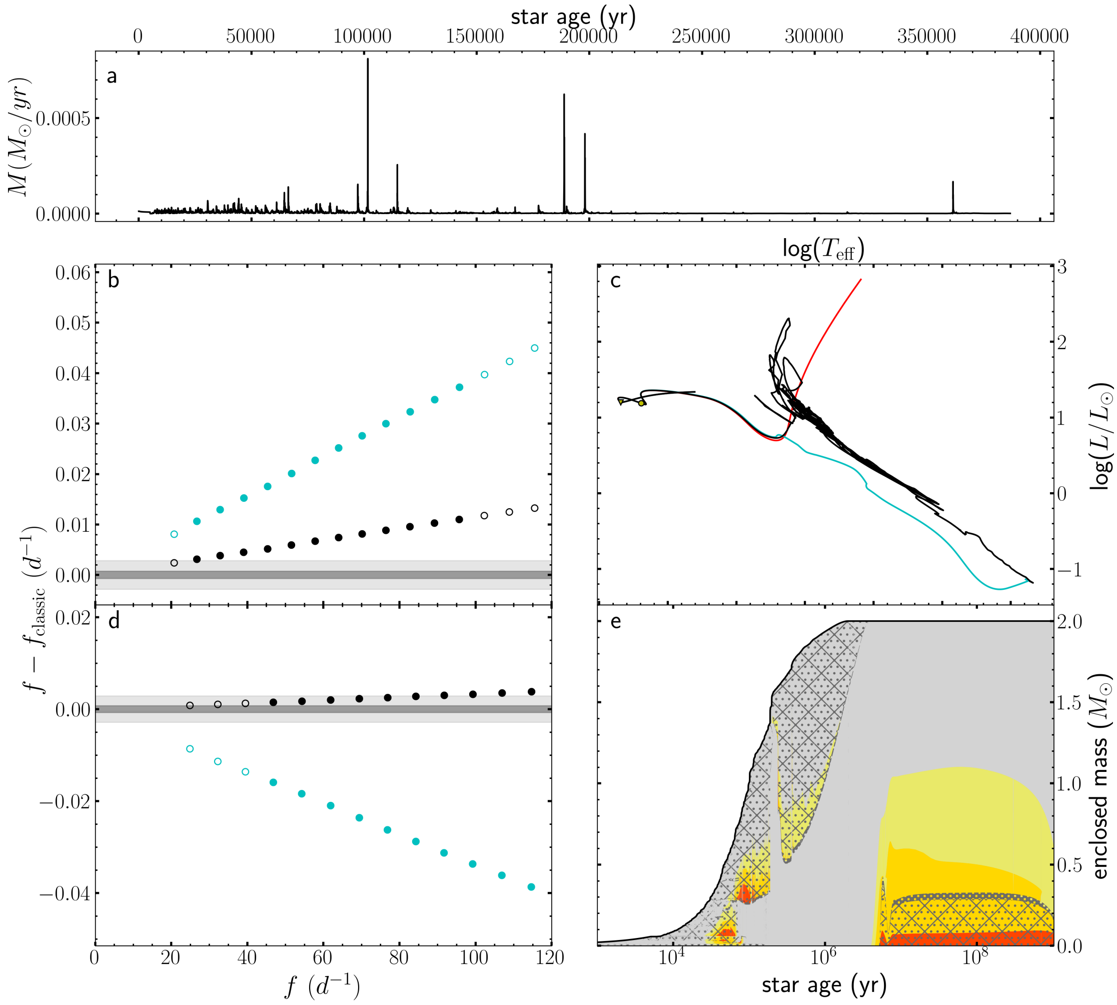

Supplementary Figure 15: Comparison between classical, constant accretion, and disk-mediated accretion model #13 for the standard input physics. a The adopted accretion rate as a function of star age. **b** The frequency difference of $l = 1, m = 1$ modes as a function of the pulsation frequency for the predefined pre-main sequence stage. The black (turquoise) circles correspond to differences between the disk-mediated accretion (constant accretion) model and the classical model. Unstable modes are filled, while stable modes are depicted as open circles. The grey areas mark the Rayleigh limit corresponding to 4-year Kepler (dark grey) and 357-days TESS light curves (light grey). **c** The evolution of the classical model (red), the constant accretion model (turquoise) and the disk-mediated accretion model (black) in the Hertzsprung-Russell. **d** Same as panel b but for the evolution model at the ZAMS. **e** Kippenhahn diagram of the evolution (same style as in Figure 1).

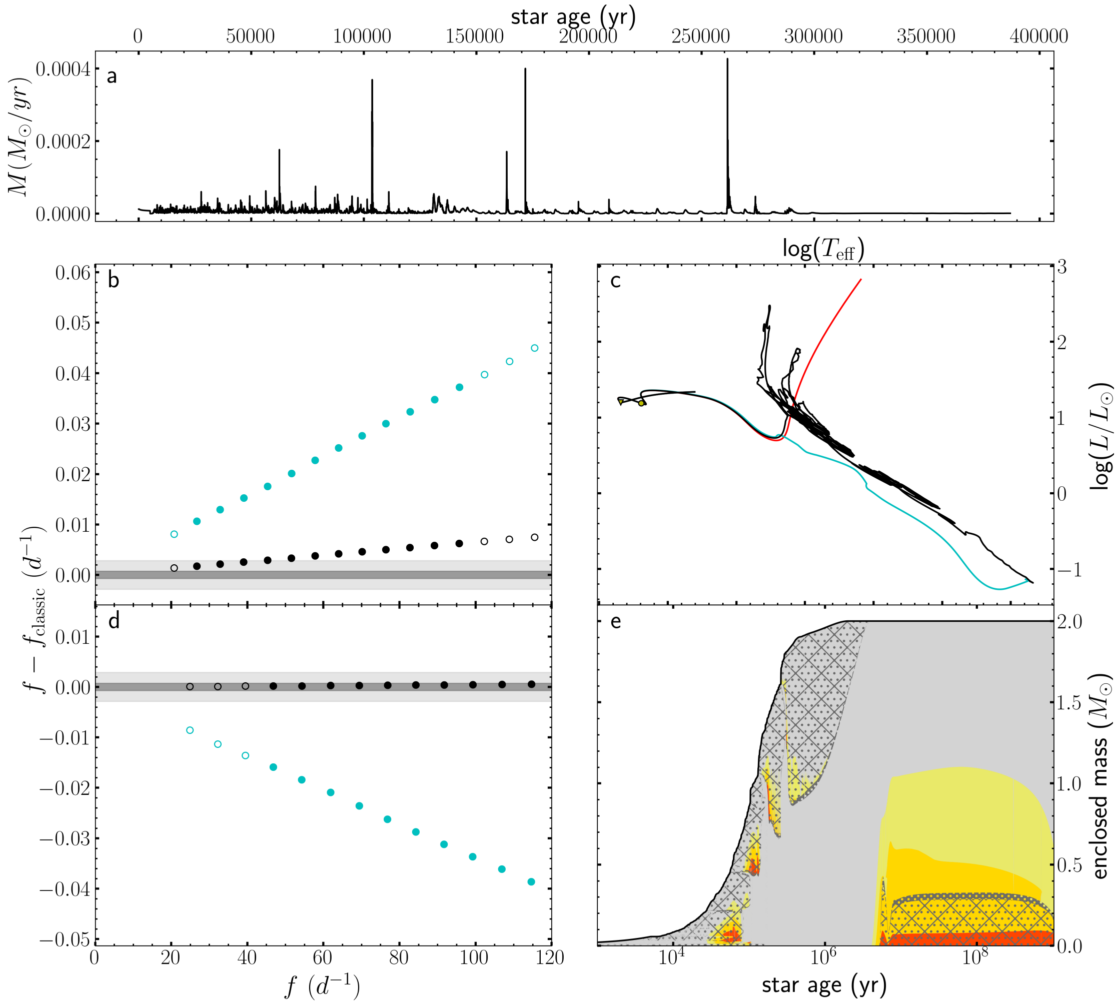

Supplementary Figure 16: Comparison between classical, constant accretion, and disk-mediated accretion model #14 for the standard input physics. a The adopted accretion rate as a function of star age. **b** The frequency difference of $l = 1, m = 1$ modes as a function of the pulsation frequency for the predefined pre-main sequence stage. The black (turquoise) circles correspond to differences between the disk-mediated accretion (constant accretion) model and the classical model. Unstable modes are filled, while stable modes are depicted as open circles. The grey areas mark the Rayleigh limit corresponding to 4-year Kepler (dark grey) and 357-days TESS light curves (light grey). **c** The evolution of the classical model (red), the constant accretion model (turquoise) and the disk-mediated accretion model (black) in the Hertzsprung-Russell. **d** Same as panel b but for the evolution model at the ZAMS. **e** Kippenhahn diagram of the evolution (same style as in Figure 1).

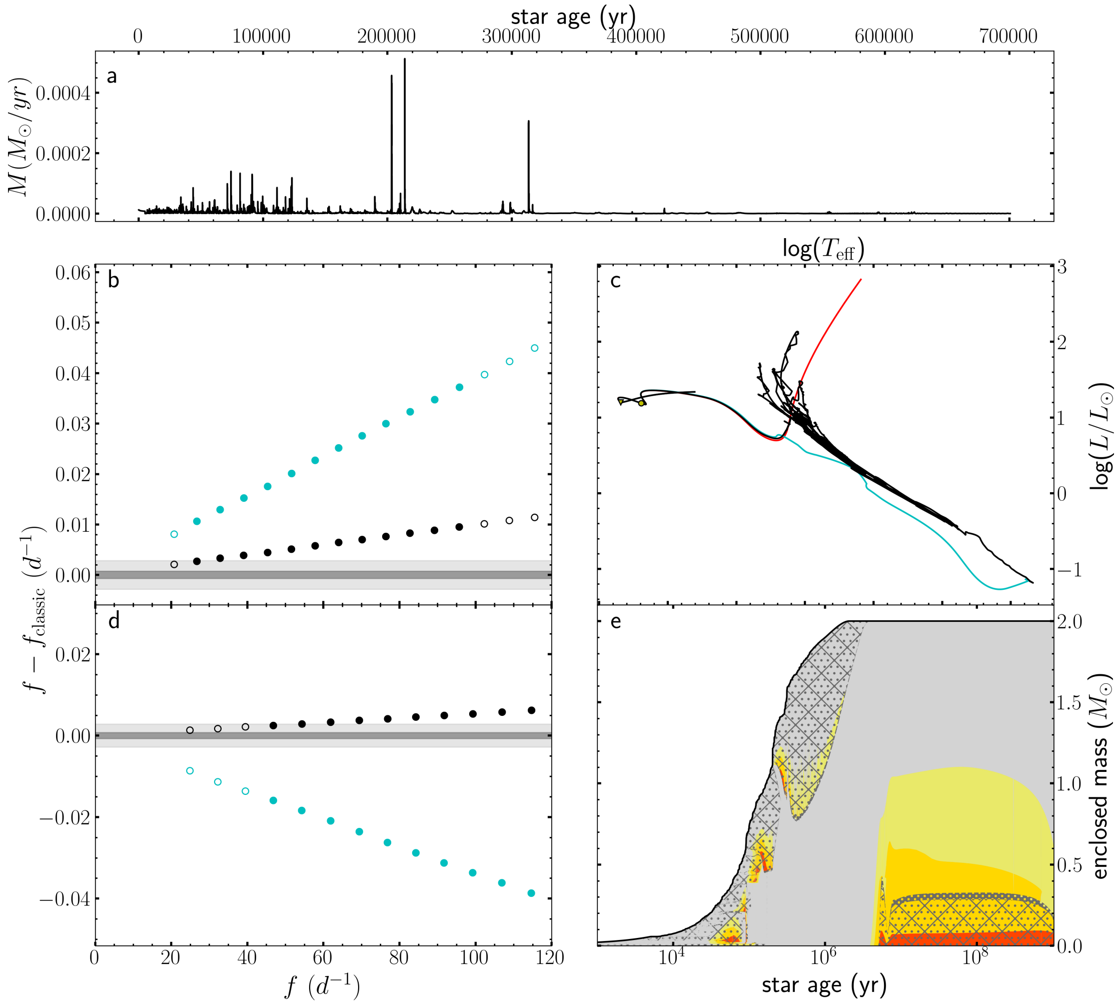

Supplementary Figure 17: Comparison between classical, constant accretion, and disk-mediated accretion model #15 for the standard input physics. **a** The adopted accretion rate as a function of star age. **b** The frequency difference of $l = 1, m = 1$ modes as a function of the pulsation frequency for the predefined pre-main sequence stage. The black (turquoise) circles correspond to differences between the disk-mediated accretion (constant accretion) model and the classical model. Unstable modes are filled, while stable modes are depicted as open circles. The grey areas mark the Rayleigh limit corresponding to 4-year Kepler (dark grey) and 357-days TESS light curves (light grey). **c** The evolution of the classical model (red), the constant accretion model (turquoise) and the disk-mediated accretion model (black) in the Hertzsprung-Russell. **d** Same as panel b but for the evolution model at the ZAMS. **e** Kippenhahn diagram of the evolution (same style as in Figure 1).

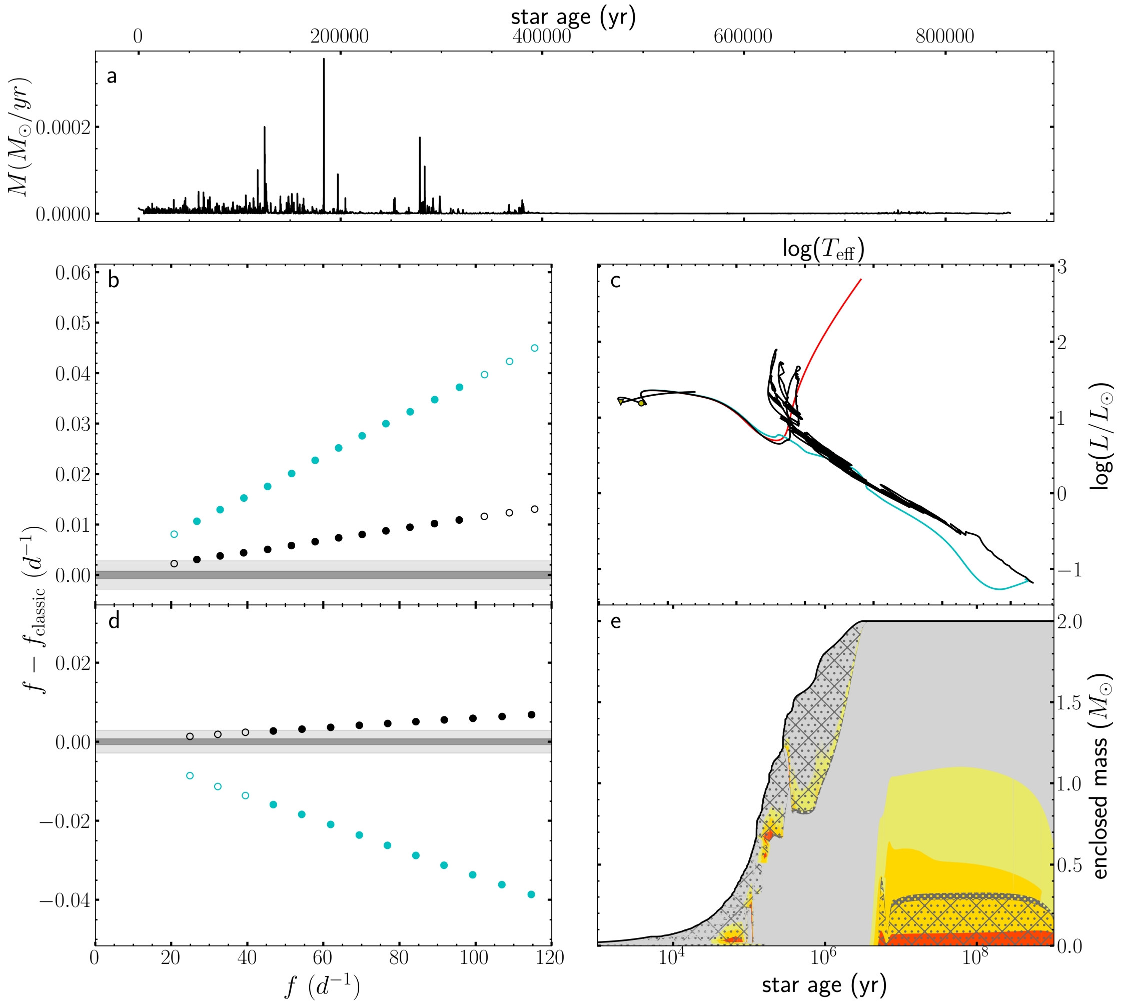

Supplementary Figure 18: Comparison between classical, constant accretion, and disk-mediated accretion model #16 for the standard input physics. a The adopted accretion rate as a function of star age. **b** The frequency difference of $l = 1, m = 1$ modes as a function of the pulsation frequency for the predefined pre-main sequence stage. The black (turquoise) circles correspond to differences between the disk-mediated accretion (constant accretion) model and the classical model. Unstable modes are filled, while stable modes are depicted as open circles. The grey areas mark the Rayleigh limit corresponding to 4-year Kepler (dark grey) and 357-days TESS light curves (light grey). **c** The evolution of the classical model (red), the constant accretion model (turquoise) and the disk-mediated accretion model (black) in the Hertzsprung-Russell. **d** Same as panel b but for the evolution model at the ZAMS. **e** Kippenhahn diagram of the evolution (same style as in Figure 1).

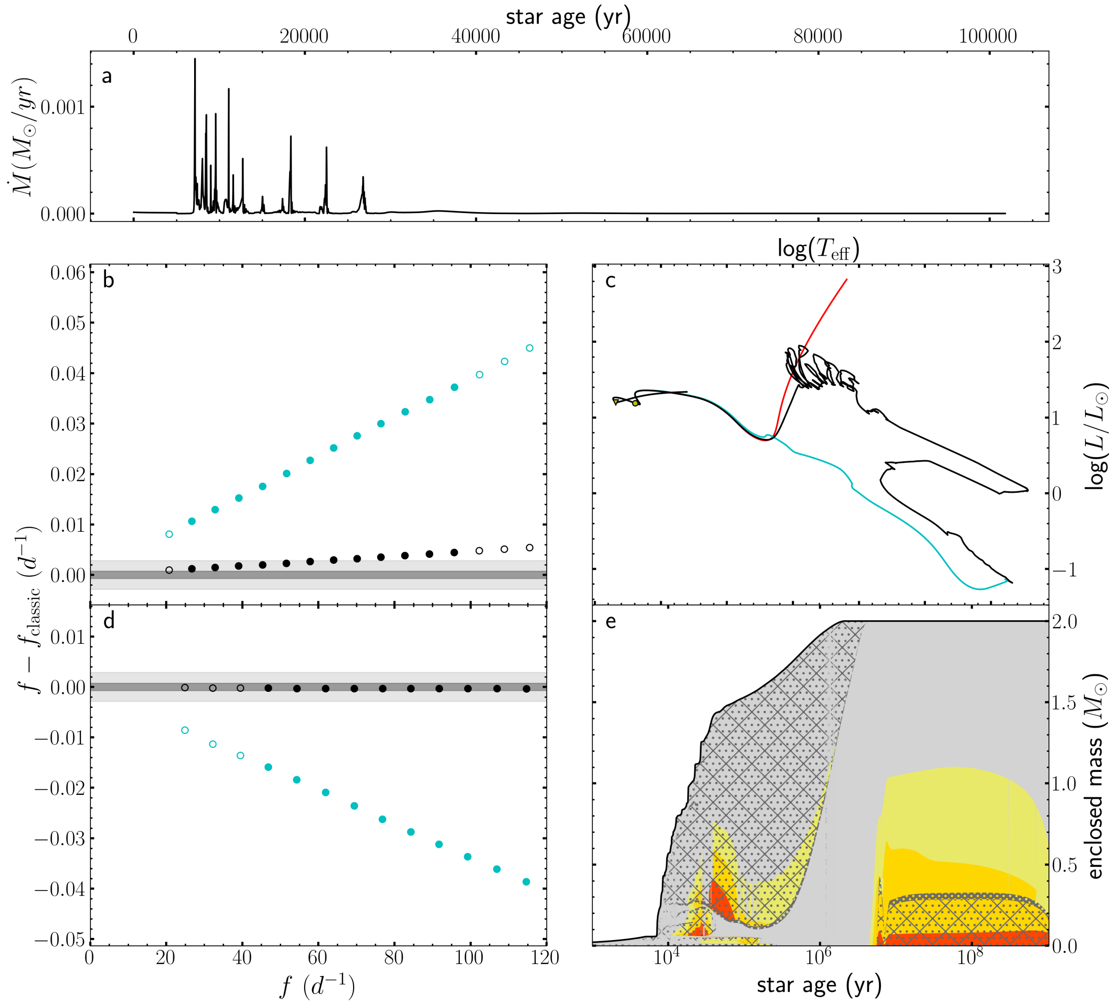

Supplementary Figure 19: Comparison between classical, constant accretion, and disk-mediated accretion model #18 for the standard input physics. a The adopted accretion rate as a function of star age. **b** The frequency difference of $l = 1, m = 1$ modes as a function of the pulsation frequency for the predefined pre-main sequence stage. The black (turquoise) circles correspond to differences between the disk-mediated accretion (constant accretion) model and the classical model. Unstable modes are filled, while stable modes are depicted as open circles. The grey areas mark the Rayleigh limit corresponding to 4-year Kepler (dark grey) and 357-days TESS light curves (light grey). **c** The evolution of the classical model (red), the constant accretion model (turquoise) and the disk-mediated accretion model (black) in the Hertzsprung-Russell. **d** Same as panel b but for the evolution model at the ZAMS. **e** Kippenhahn diagram of the evolution (same style as in Figure 1).

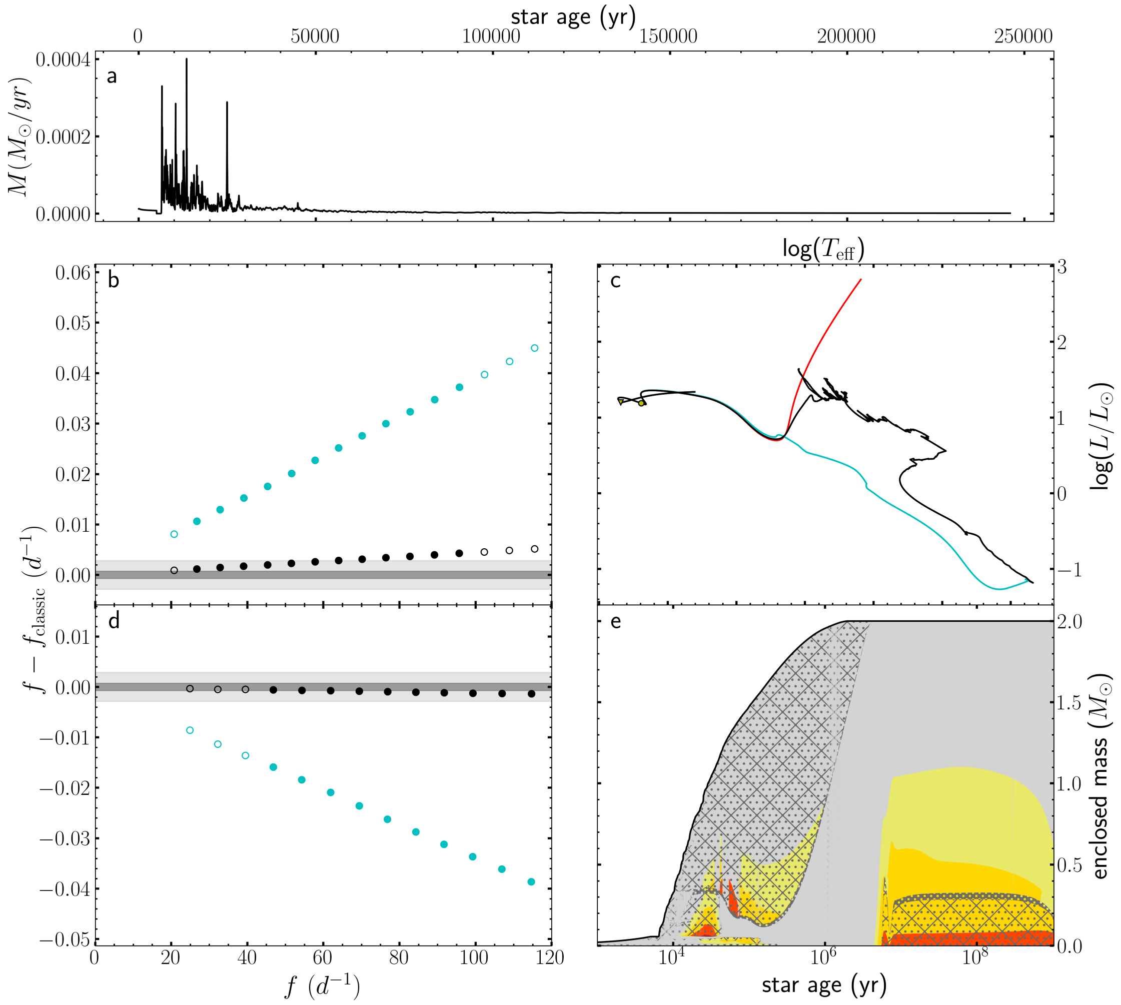

Supplementary Figure 20: Comparison between classical, constant accretion, and disk-mediated accretion model #19 for the standard input physics. a The adopted accretion rate as a function of star age. **b** The frequency difference of $l = 1, m = 1$ modes as a function of the pulsation frequency for the predefined pre-main sequence stage. The black (turquoise) circles correspond to differences between the disk-mediated accretion (constant accretion) model and the classical model. Unstable modes are filled, while stable modes are depicted as open circles. The grey areas mark the Rayleigh limit corresponding to 4-year Kepler (dark grey) and 357-days TESS light curves (light grey). **c** The evolution of the classical model (red), the constant accretion model (turquoise) and the disk-mediated accretion model (black) in the Hertzsprung-Russell. **d** Same as panel b but for the evolution model at the ZAMS. **e** Kippenhahn diagram of the evolution (same style as in Figure 1).

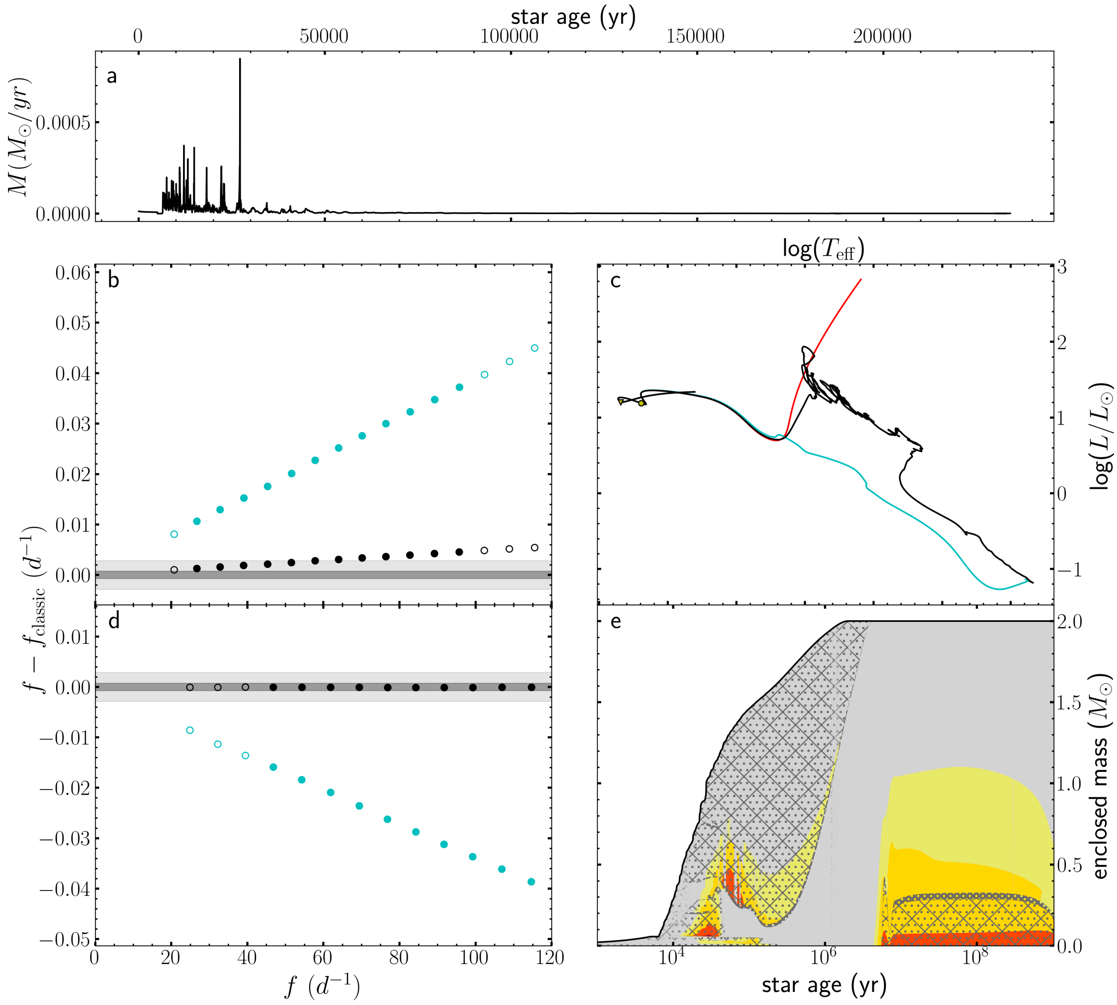

Supplementary Figure 21: Comparison between classical, constant accretion, and disk-mediated accretion model #20 for the standard input physics. a The adopted accretion rate as a function of star age. **b** The frequency difference of $l = 1, m = 1$ modes as a function of the pulsation frequency for the predefined pre-main sequence stage. The black (turquoise) circles correspond to differences between the disk-mediated accretion (constant accretion) model and the classical model. Unstable modes are filled, while stable modes are depicted as open circles. The grey areas mark the Rayleigh limit corresponding to 4-year Kepler (dark grey) and 357-days TESS light curves (light grey). **c** The evolution of the classical model (red), the constant accretion model (turquoise) and the disk-mediated accretion model (black) in the Hertzsprung-Russell. **d** Same as panel b but for the evolution model at the ZAMS. **e** Kippenhahn diagram of the evolution (same style as in Figure 1).

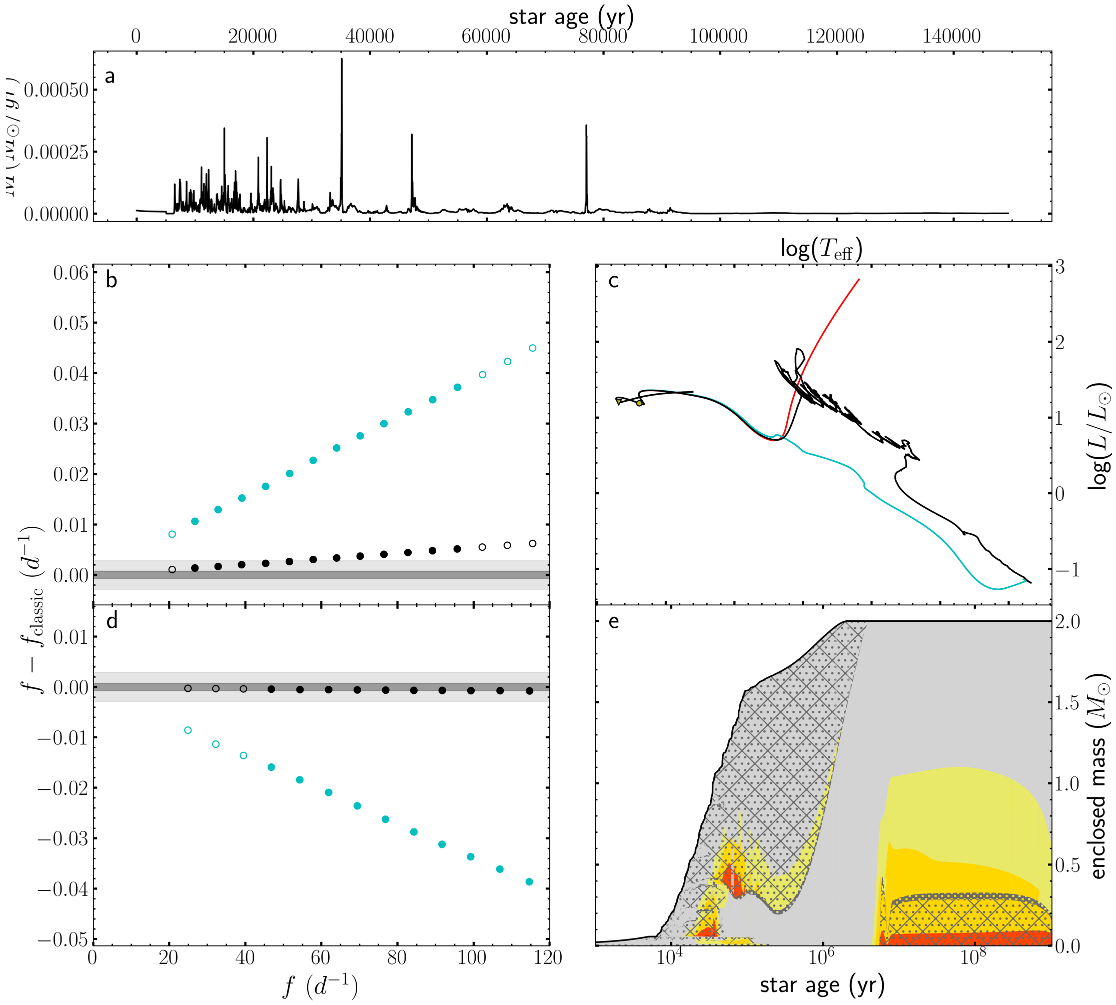

Supplementary Figure 22: Comparison between classical, constant accretion, and disk-mediated accretion model #21 for the standard input physics. a The adopted accretion rate as a function of star age. **b** The frequency difference of $l = 1, m = 1$ modes as a function of the pulsation frequency for the predefined pre-main sequence stage. The black (turquoise) circles correspond to differences between the disk-mediated accretion (constant accretion) model and the classical model. Unstable modes are filled, while stable modes are depicted as open circles. The grey areas mark the Rayleigh limit corresponding to 4-year Kepler (dark grey) and 357-days TESS light curves (light grey). **c** The evolution of the classical model (red), the constant accretion model (turquoise) and the disk-mediated accretion model (black) in the Hertzsprung-Russell. **d** Same as panel b but for the evolution model at the ZAMS. **e** Kippenhahn diagram of the evolution (same style as in Figure 1).

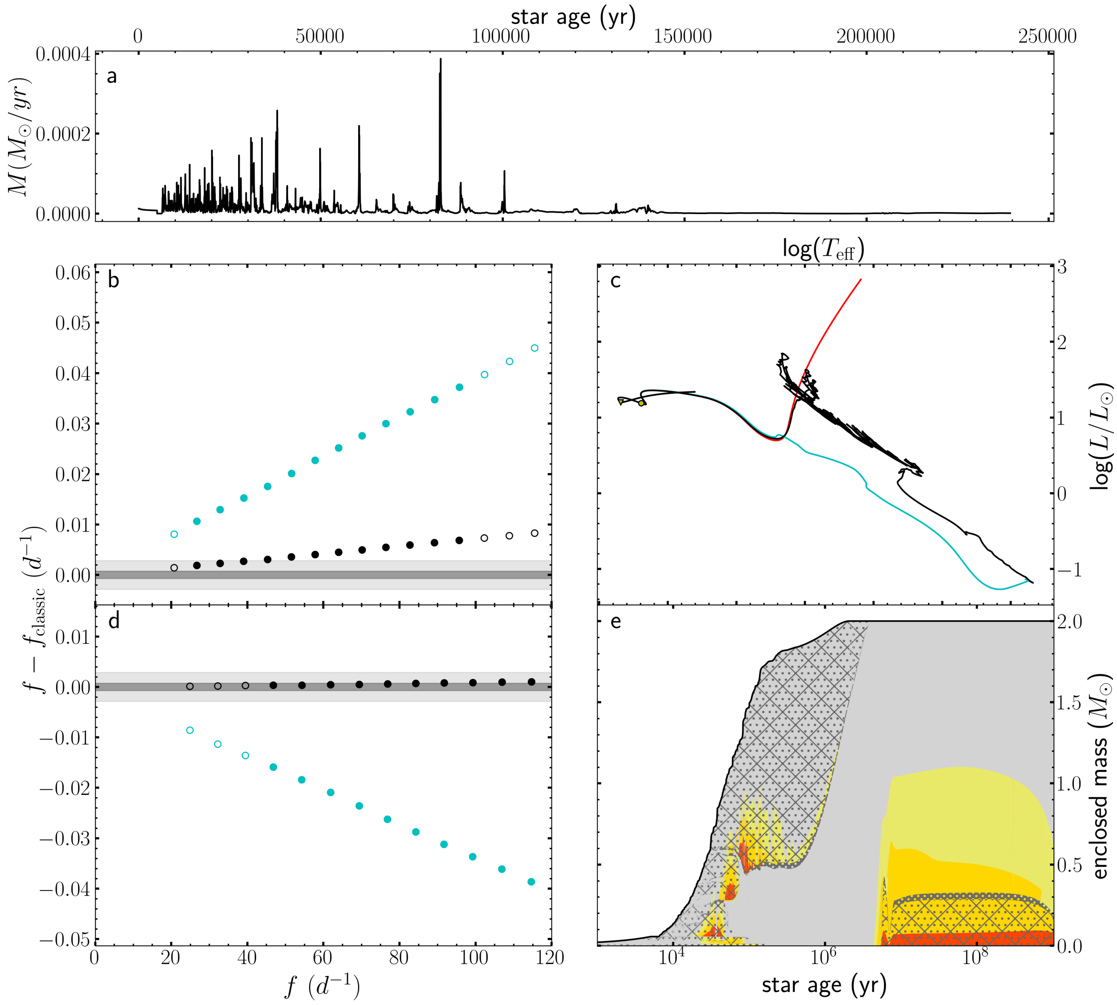

Supplementary Figure 23: Comparison between classical, constant accretion, and disk-mediated accretion model #22 for the standard input physics. **a** The adopted accretion rate as a function of star age. **b** The frequency difference of $l = 1, m = 1$ modes as a function of the pulsation frequency for the predefined pre-main sequence stage. The black (turquoise) circles correspond to differences between the disk-mediated accretion (constant accretion) model and the classical model. Unstable modes are filled, while stable modes are depicted as open circles. The grey areas mark the Rayleigh limit corresponding to 4-year Kepler (dark grey) and 357-days TESS light curves (light grey). **c** The evolution of the classical model (red), the constant accretion model (turquoise) and the disk-mediated accretion model (black) in the Hertzsprung-Russell. **d** Same as panel b but for the evolution model at the ZAMS. **e** Kippenhahn diagram of the evolution (same style as in Figure 1).

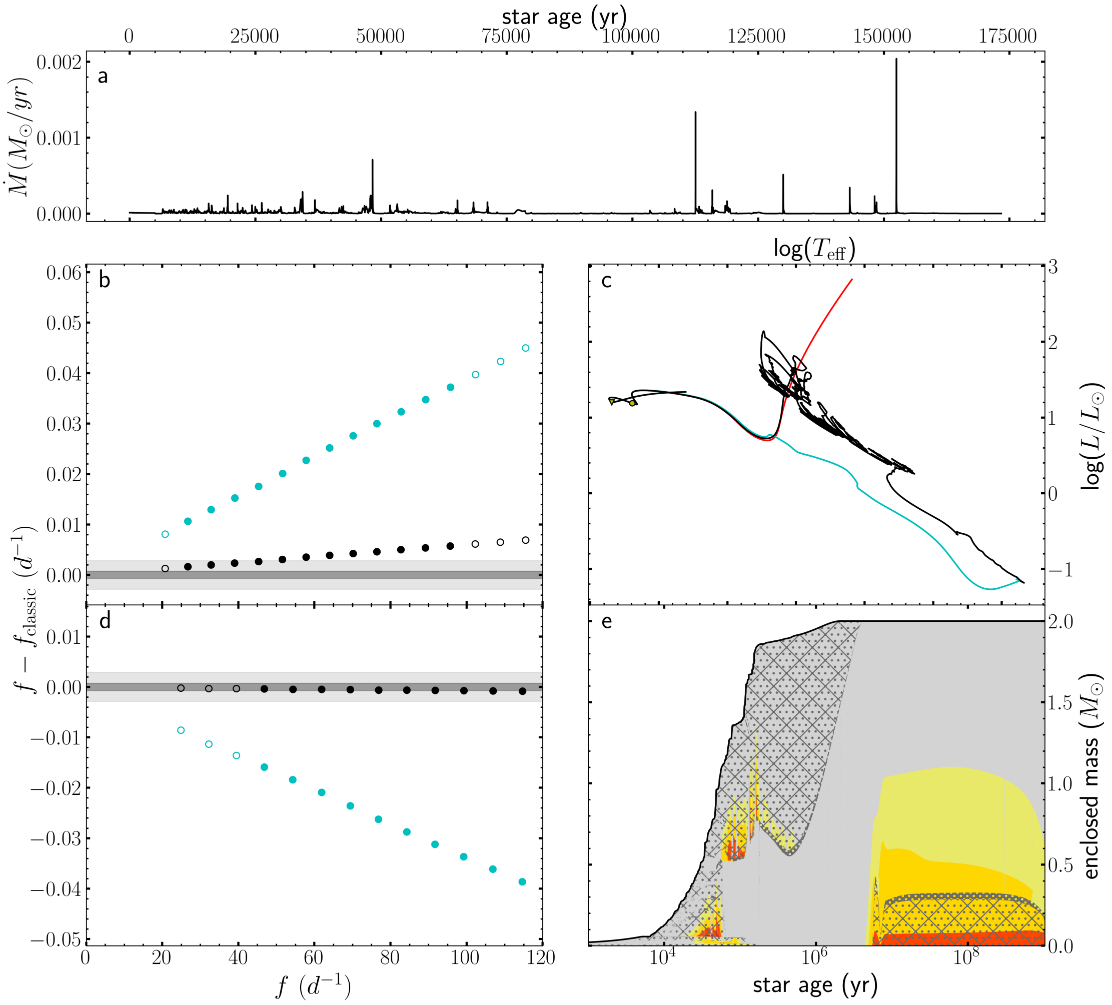

Supplementary Figure 24: Comparison between classical, constant accretion, and disk-mediated accretion model #23 for the standard input physics. a The adopted accretion rate as a function of star age. **b** The frequency difference of $l = 1, m = 1$ modes as a function of the pulsation frequency for the predefined pre-main sequence stage. The black (turquoise) circles correspond to differences between the disk-mediated accretion (constant accretion) model and the classical model. Unstable modes are filled, while stable modes are depicted as open circles. The grey areas mark the Rayleigh limit corresponding to 4-year Kepler (dark grey) and 357-days TESS light curves (light grey). **c** The evolution of the classical model (red), the constant accretion model (turquoise) and the disk-mediated accretion model (black) in the Hertzsprung-Russell. **d** Same as panel b but for the evolution model at the ZAMS. **e** Kippenhahn diagram of the evolution (same style as in Figure 1).

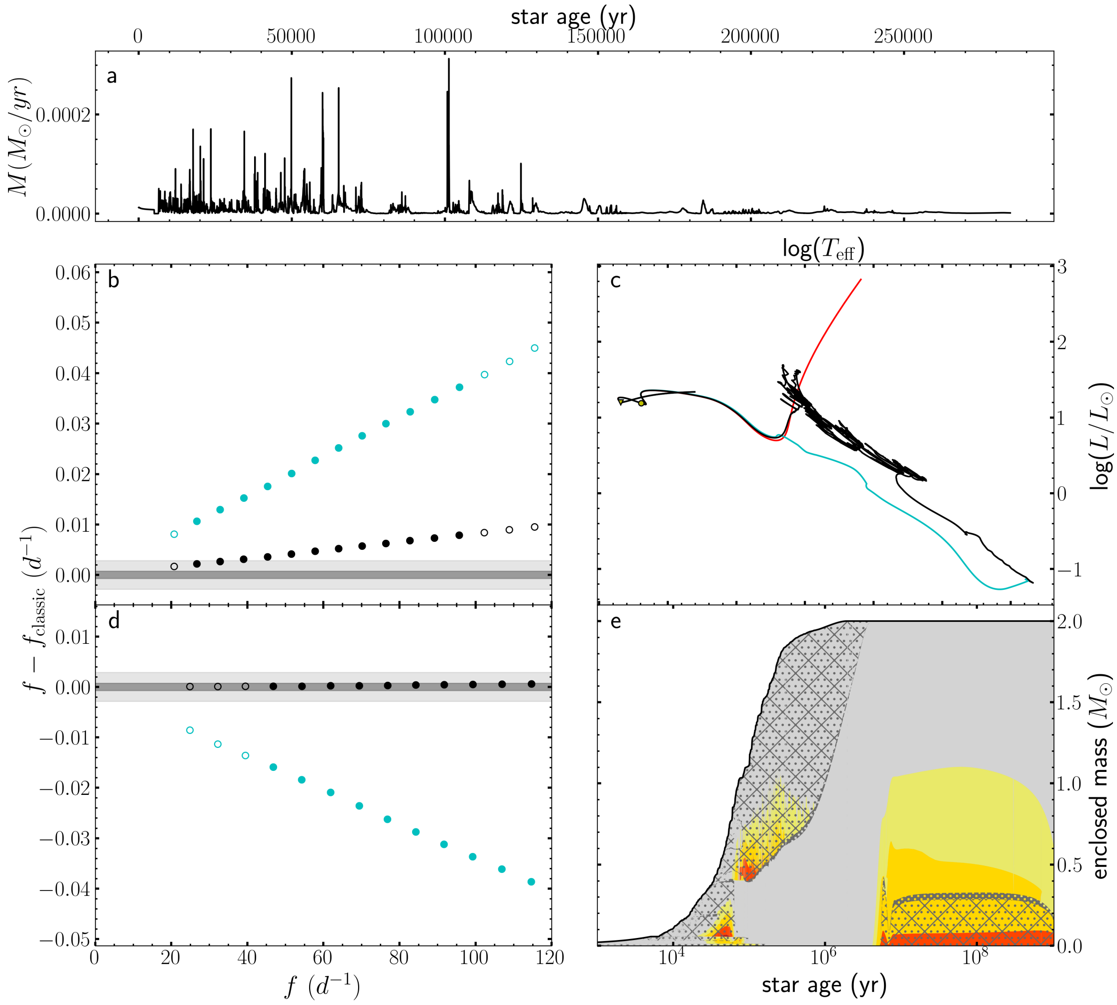

Supplementary Figure 25: Comparison between classical, constant accretion, and disk-mediated accretion model #24 for the standard input physics. a The adopted accretion rate as a function of star age. **b** The frequency difference of $l = 1, m = 1$ modes as a function of the pulsation frequency for the predefined pre-main sequence stage. The black (turquoise) circles correspond to differences between the disk-mediated accretion (constant accretion) model and the classical model. Unstable modes are filled, while stable modes are depicted as open circles. The grey areas mark the Rayleigh limit corresponding to 4-year Kepler (dark grey) and 357-days TESS light curves (light grey). **c** The evolution of the classical model (red), the constant accretion model (turquoise) and the disk-mediated accretion model (black) in the Hertzsprung-Russell. **d** Same as panel b but for the evolution model at the ZAMS. **e** Kippenhahn diagram of the evolution (same style as in Figure 1).

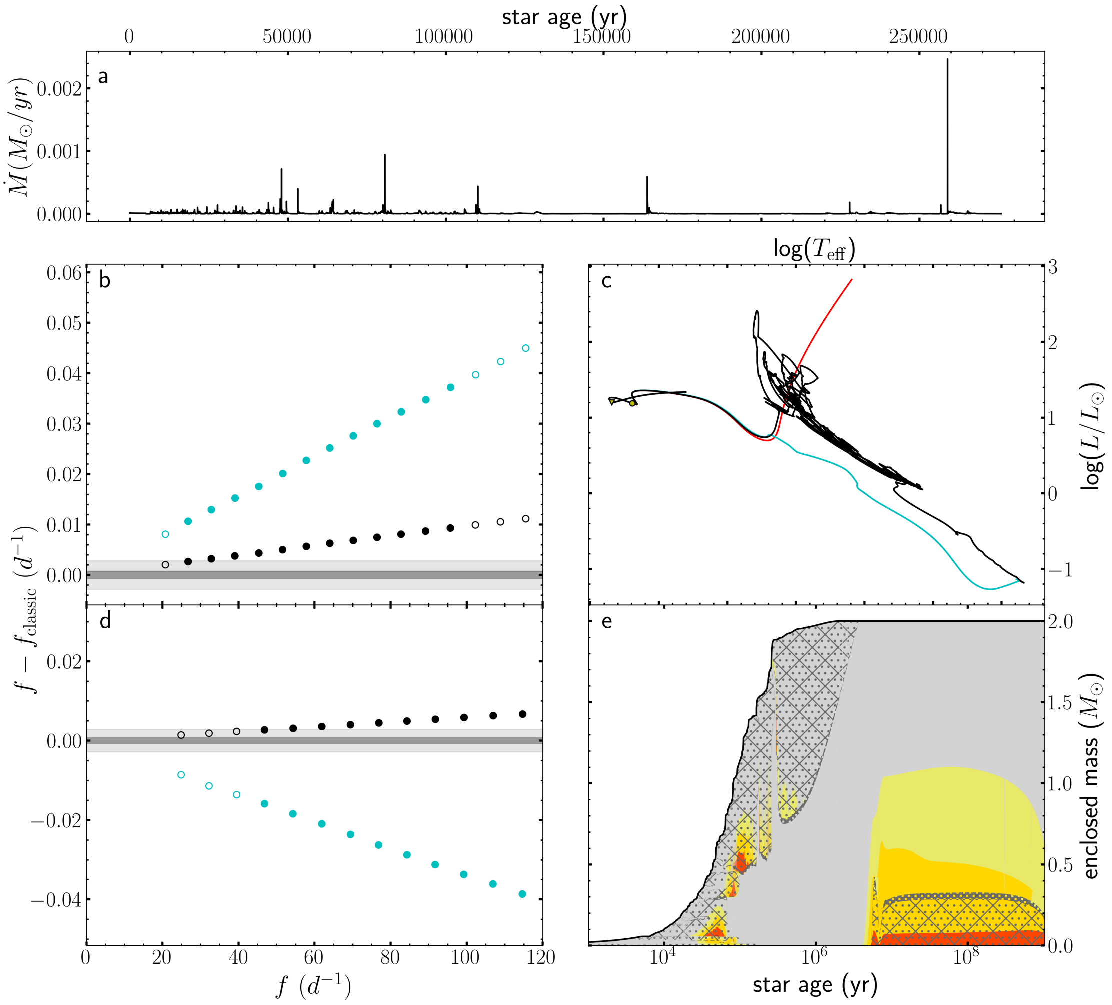

Supplementary Figure 26: Comparison between classical, constant accretion, and disk-mediated accretion model #25 for the standard input physics. **a** The adopted accretion rate as a function of star age. **b** The frequency difference of $l = 1, m = 1$ modes as a function of the pulsation frequency for the predefined pre-main sequence stage. The black (turquoise) circles correspond to differences between the disk-mediated accretion (constant accretion) model and the classical model. Unstable modes are filled, while stable modes are depicted as open circles. The grey areas mark the Rayleigh limit corresponding to 4-year Kepler (dark grey) and 357-days TESS light curves (light grey). **c** The evolution of the classical model (red), the constant accretion model (turquoise) and the disk-mediated accretion model (black) in the Hertzsprung-Russell. **d** Same as panel b but for the evolution model at the ZAMS. **e** Kippenhahn diagram of the evolution (same style as in Figure 1).

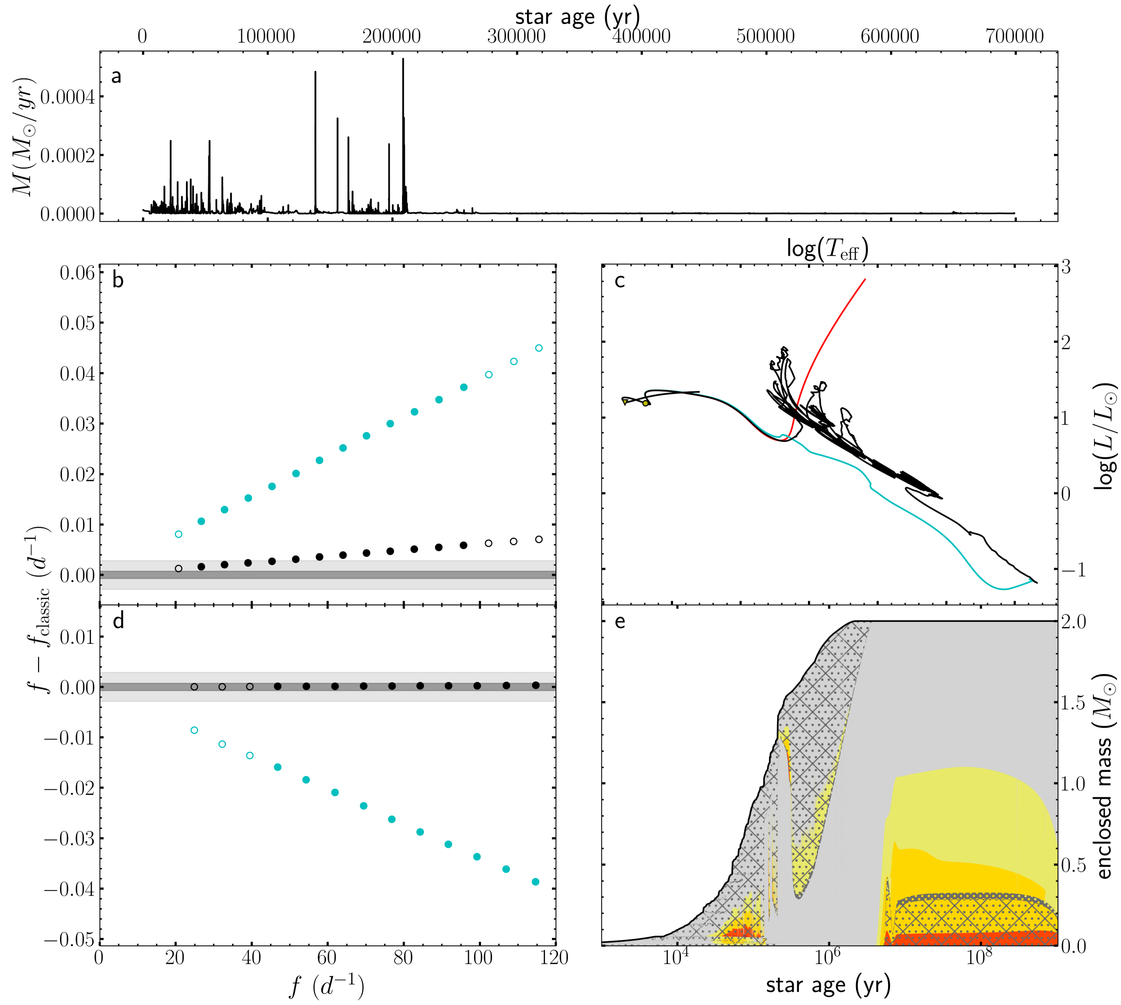

Supplementary Figure 27: Comparison between classical, constant accretion, and disk-mediated accretion model #26 for the standard input physics. a The adopted accretion rate as a function of star age. **b** The frequency difference of $l = 1, m = 1$ modes as a function of the pulsation frequency for the predefined pre-main sequence stage. The black (turquoise) circles correspond to differences between the disk-mediated accretion (constant accretion) model and the classical model. Unstable modes are filled, while stable modes are depicted as open circles. The grey areas mark the Rayleigh limit corresponding to 4-year Kepler (dark grey) and 357-days TESS light curves (light grey). **c** The evolution of the classical model (red), the constant accretion model (turquoise) and the disk-mediated accretion model (black) in the Hertzsprung-Russell. **d** Same as panel b but for the evolution model at the ZAMS. **e** Kippenhahn diagram of the evolution (same style as in Figure 1).

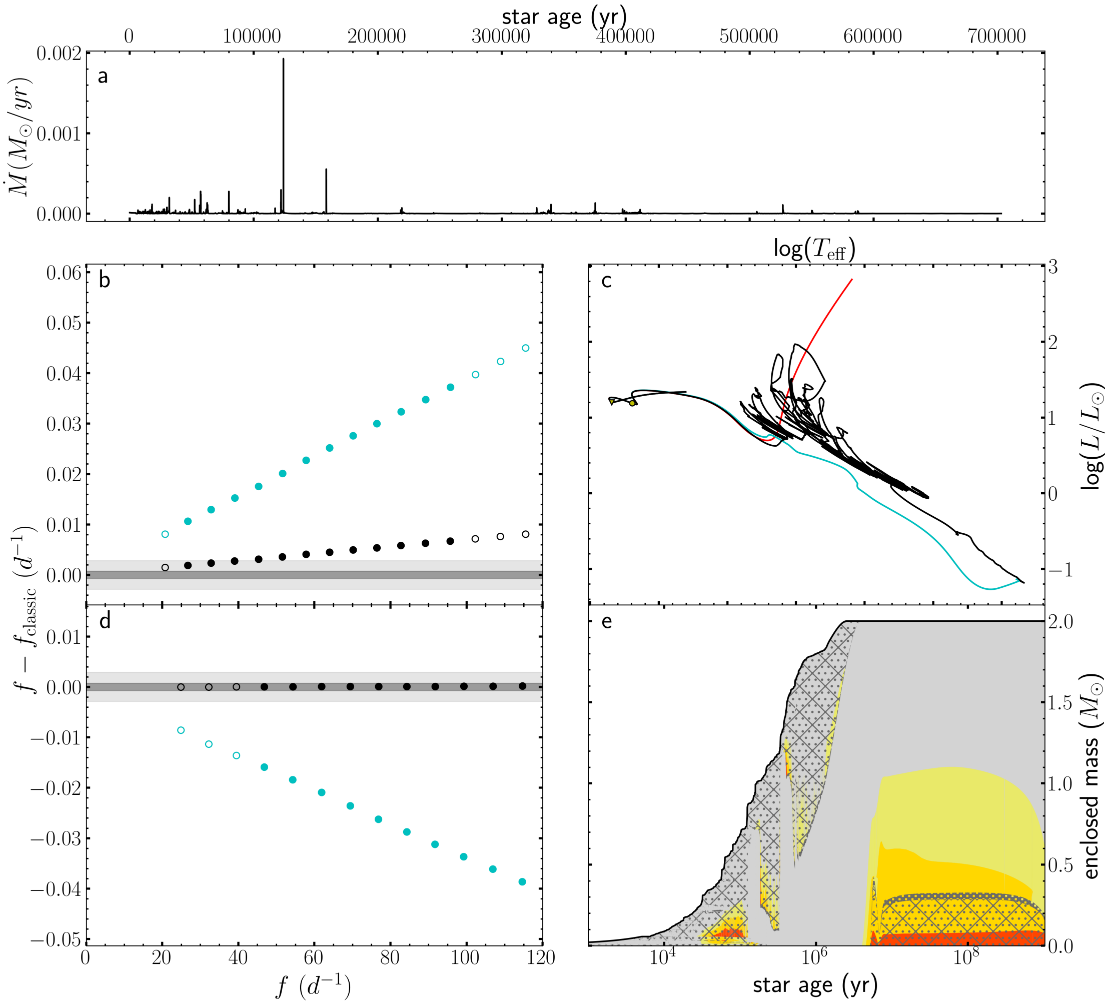

Supplementary Figure 28: Comparison between classical, constant accretion, and disk-mediated accretion model #27 for the standard input physics. a The adopted accretion rate as a function of star age. **b** The frequency difference of $l = 1, m = 1$ modes as a function of the pulsation frequency for the predefined pre-main sequence stage. The black (turquoise) circles correspond to differences between the disk-mediated accretion (constant accretion) model and the classical model. Unstable modes are filled, while stable modes are depicted as open circles. The grey areas mark the Rayleigh limit corresponding to 4-year Kepler (dark grey) and 357-days TESS light curves (light grey). **c** The evolution of the classical model (red), the constant accretion model (turquoise) and the disk-mediated accretion model (black) in the Hertzsprung-Russell. **d** Same as panel b but for the evolution model at the ZAMS. **e** Kippenhahn diagram of the evolution (same style as in Figure 1).

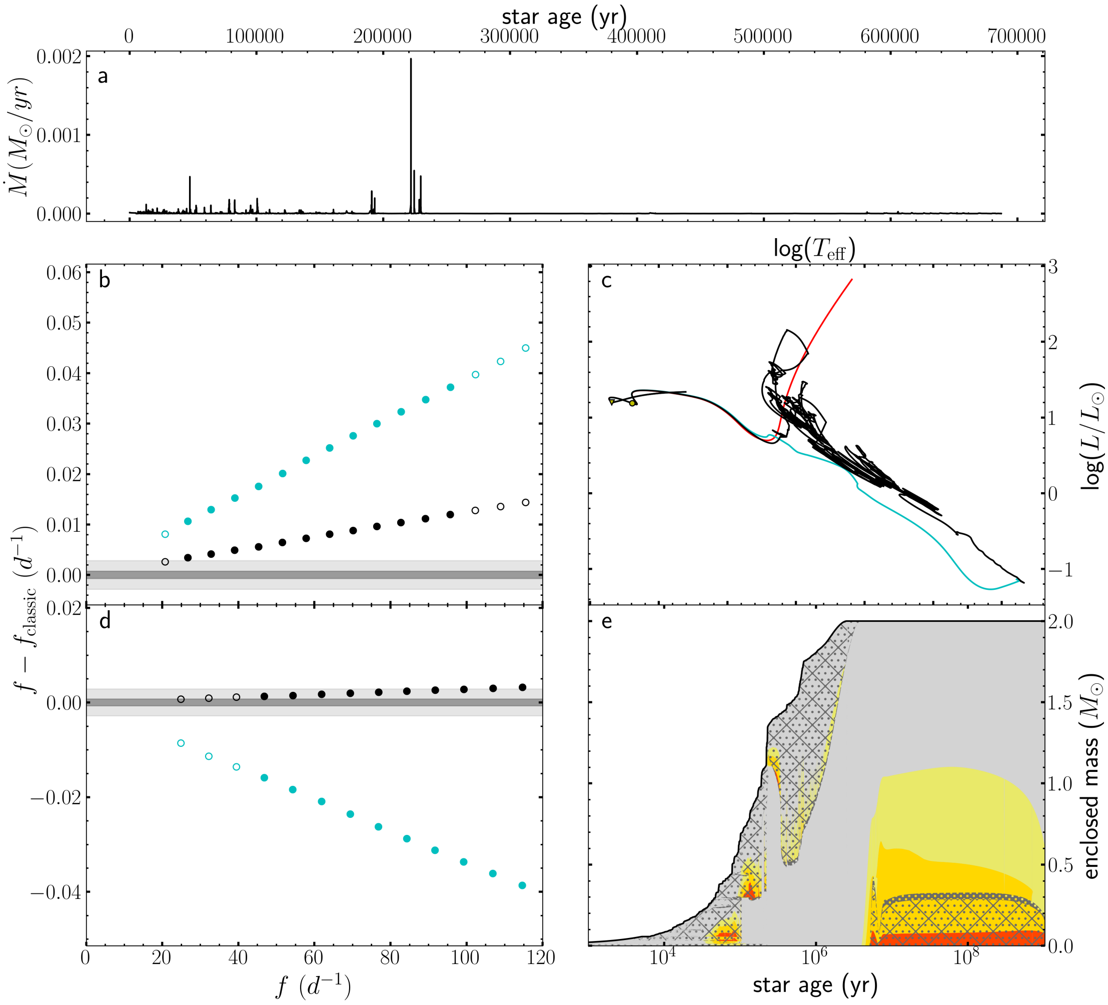

Supplementary Figure 29: Comparison between classical, constant accretion, and disk-mediated accretion model #28 for the standard input physics. a The adopted accretion rate as a function of star age. **b** The frequency difference of $l = 1, m = 1$ modes as a function of the pulsation frequency for the predefined pre-main sequence stage. The black (turquoise) circles correspond to differences between the disk-mediated accretion (constant accretion) model and the classical model. Unstable modes are filled, while stable modes are depicted as open circles. The grey areas mark the Rayleigh limit corresponding to 4-year Kepler (dark grey) and 357-days TESS light curves (light grey). **c** The evolution of the classical model (red), the constant accretion model (turquoise) and the disk-mediated accretion model (black) in the Hertzsprung-Russell. **d** Same as panel b but for the evolution model at the ZAMS. **e** Kippenhahn diagram of the evolution (same style as in Figure 1).

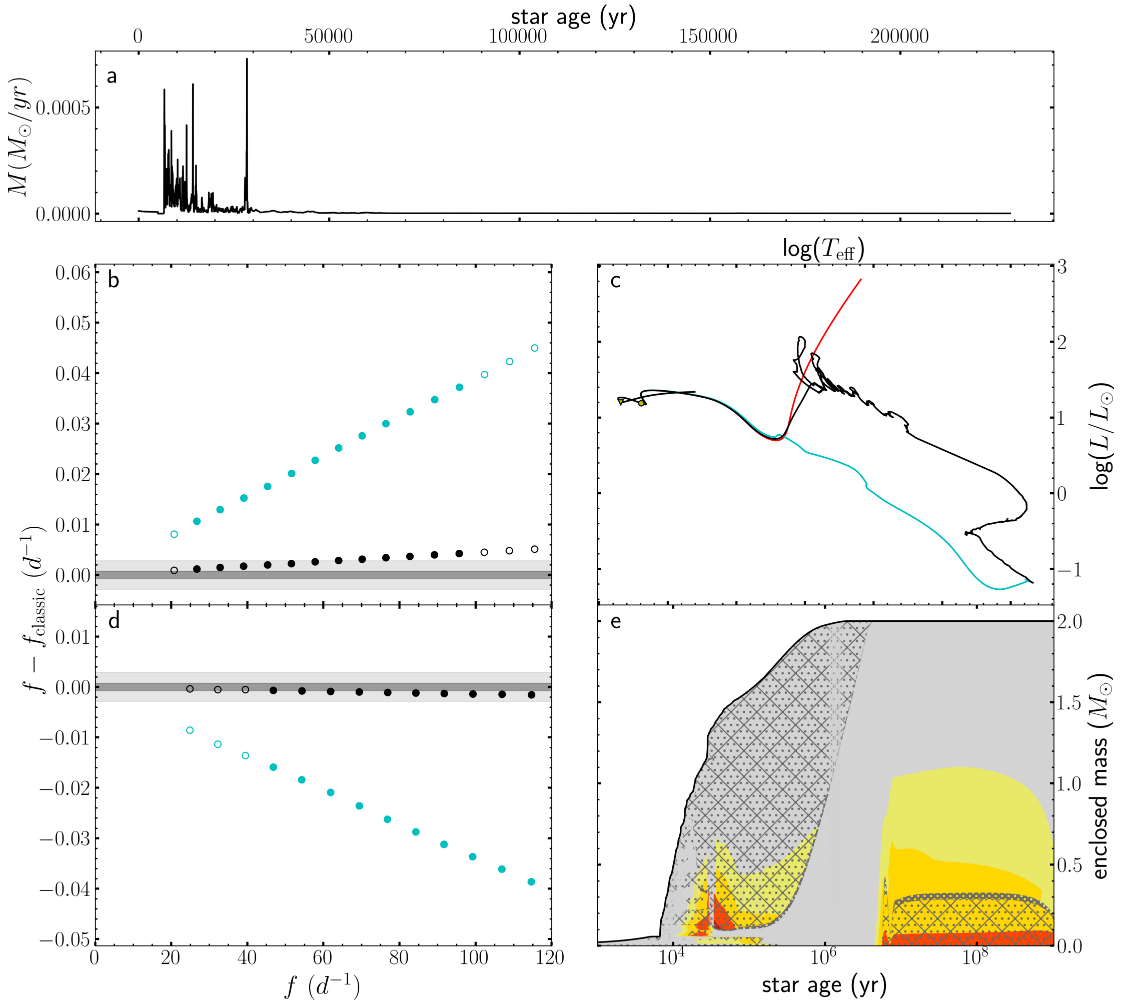

Supplementary Figure 30: Comparison between classical, constant accretion, and disk-mediated accretion model #29 for the standard input physics. a The adopted accretion rate as a function of star age. **b** The frequency difference of $l = 1, m = 1$ modes as a function of the pulsation frequency for the predefined pre-main sequence stage. The black (turquoise) circles correspond to differences between the disk-mediated accretion (constant accretion) model and the classical model. Unstable modes are filled, while stable modes are depicted as open circles. The grey areas mark the Rayleigh limit corresponding to 4-year Kepler (dark grey) and 357-days TESS light curves (light grey). **c** The evolution of the classical model (red), the constant accretion model (turquoise) and the disk-mediated accretion model (black) in the Hertzsprung-Russell. **d** Same as panel b but for the evolution model at the ZAMS. **e** Kippenhahn diagram of the evolution (same style as in Figure 1).

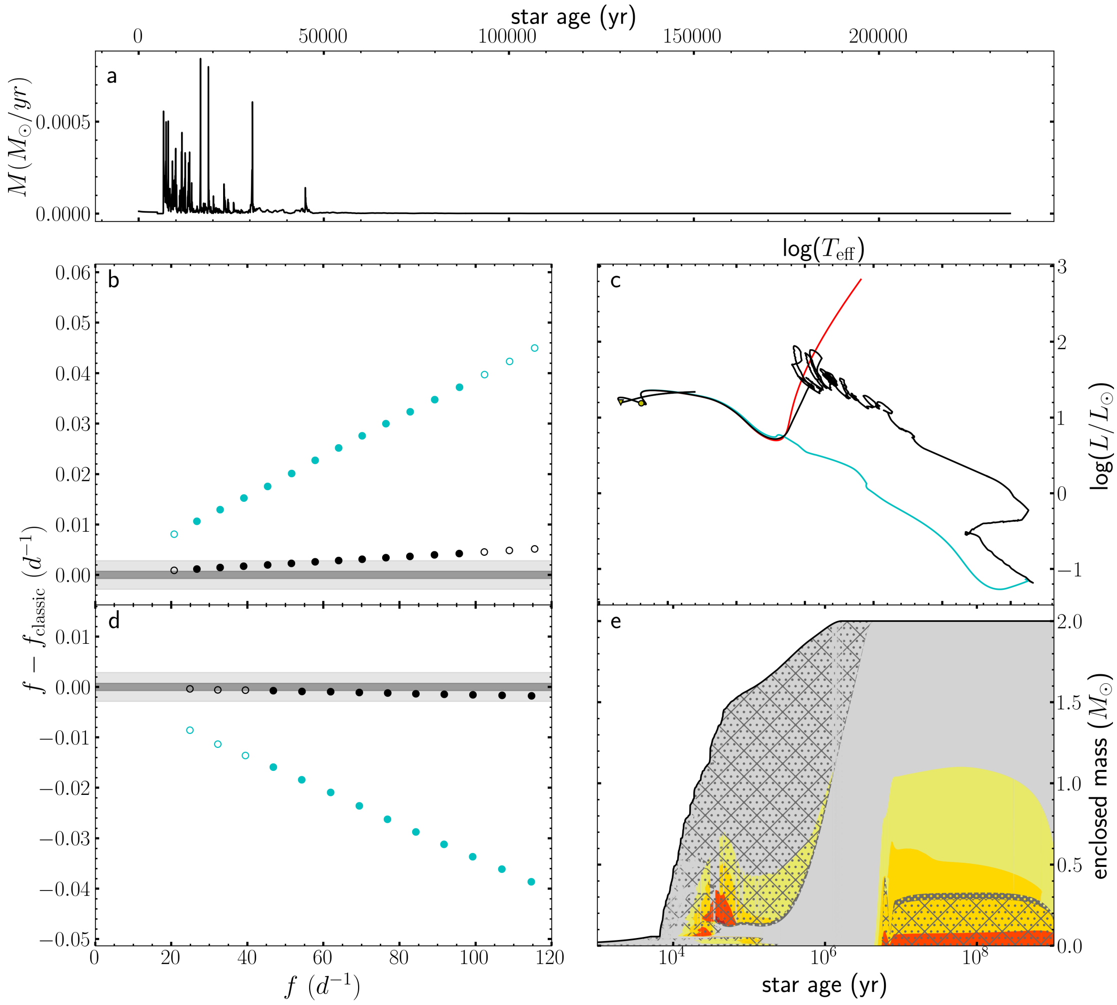

Supplementary Figure 31: Comparison between classical, constant accretion, and disk-mediated accretion model #30 for the standard input physics. a The adopted accretion rate as a function of star age. **b** The frequency difference of $l = 1, m = 1$ modes as a function of the pulsation frequency for the predefined pre-main sequence stage. The black (turquoise) circles correspond to differences between the disk-mediated accretion (constant accretion) model and the classical model. Unstable modes are filled, while stable modes are depicted as open circles. The grey areas mark the Rayleigh limit corresponding to 4-year Kepler (dark grey) and 357-days TESS light curves (light grey). **c** The evolution of the classical model (red), the constant accretion model (turquoise) and the disk-mediated accretion model (black) in the Hertzsprung-Russell. **d** Same as panel b but for the evolution model at the ZAMS. **e** Kippenhahn diagram of the evolution (same style as in Figure 1).

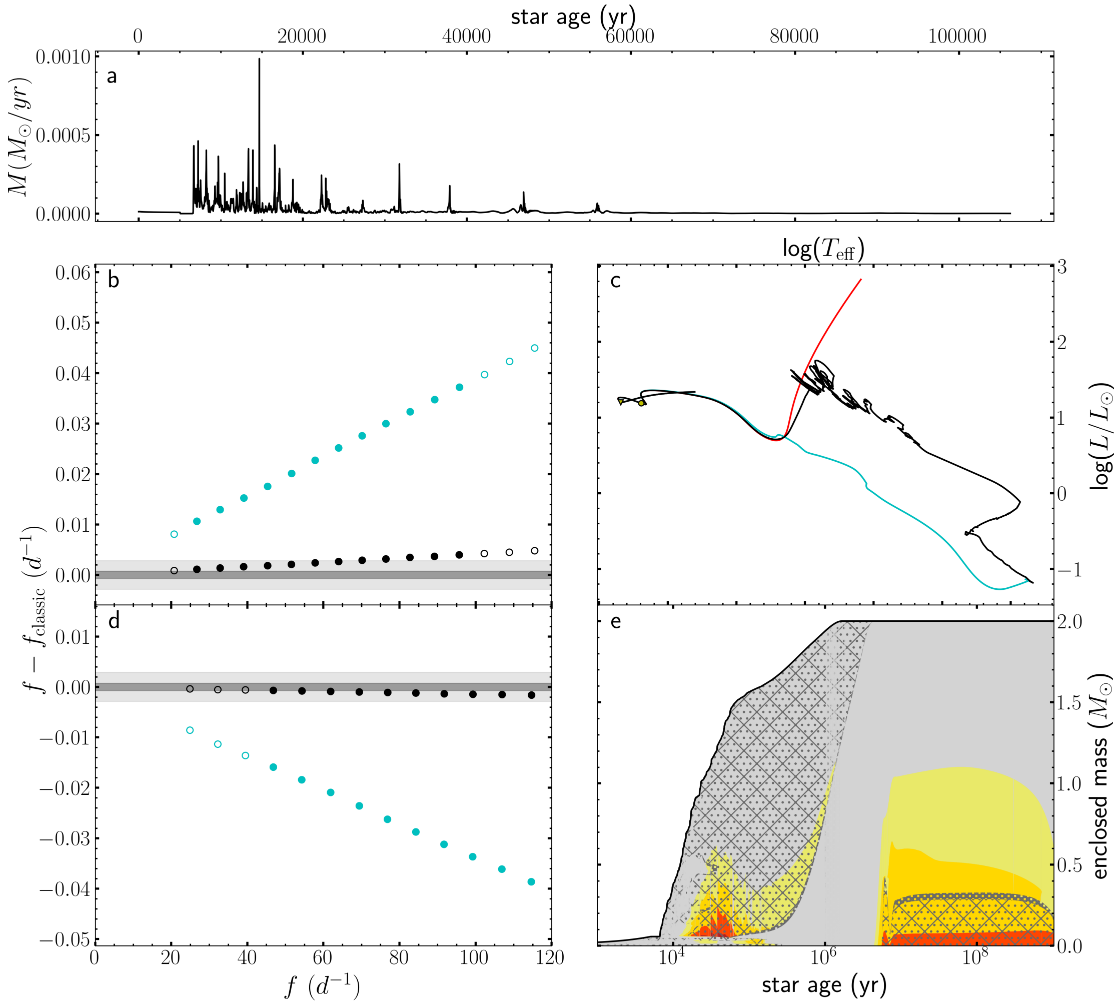

Supplementary Figure 32: Comparison between classical, constant accretion, and disk-mediated accretion model #31 for the standard input physics. a The adopted accretion rate as a function of star age. **b** The frequency difference of $l = 1, m = 1$ modes as a function of the pulsation frequency for the predefined pre-main sequence stage. The black (turquoise) circles correspond to differences between the disk-mediated accretion (constant accretion) model and the classical model. Unstable modes are filled, while stable modes are depicted as open circles. The grey areas mark the Rayleigh limit corresponding to 4-year Kepler (dark grey) and 357-days TESS light curves (light grey). **c** The evolution of the classical model (red), the constant accretion model (turquoise) and the disk-mediated accretion model (black) in the Hertzsprung-Russell. **d** Same as panel b but for the evolution model at the ZAMS. **e** Kippenhahn diagram of the evolution (same style as in Figure 1).

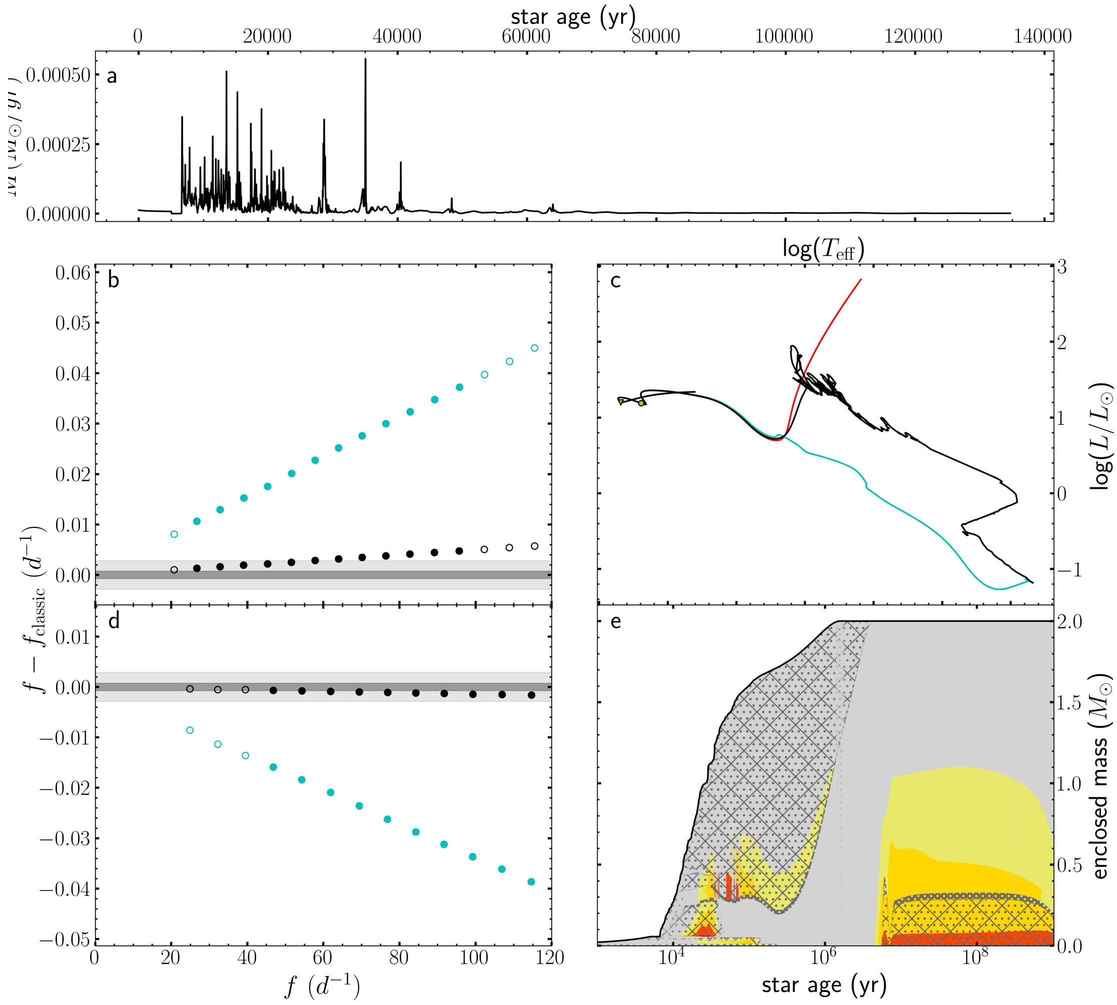

Supplementary Figure 33: Comparison between classical, constant accretion, and disk-mediated accretion model #32 for the standard input physics. a The adopted accretion rate as a function of star age. **b** The frequency difference of $l = 1, m = 1$ modes as a function of the pulsation frequency for the predefined pre-main sequence stage. The black (turquoise) circles correspond to differences between the disk-mediated accretion (constant accretion) model and the classical model. Unstable modes are filled, while stable modes are depicted as open circles. The grey areas mark the Rayleigh limit corresponding to 4-year Kepler (dark grey) and 357-days TESS light curves (light grey). **c** The evolution of the classical model (red), the constant accretion model (turquoise) and the disk-mediated accretion model (black) in the Hertzsprung-Russell. **d** Same as panel b but for the evolution model at the ZAMS. **e** Kippenhahn diagram of the evolution (same style as in Figure 1).

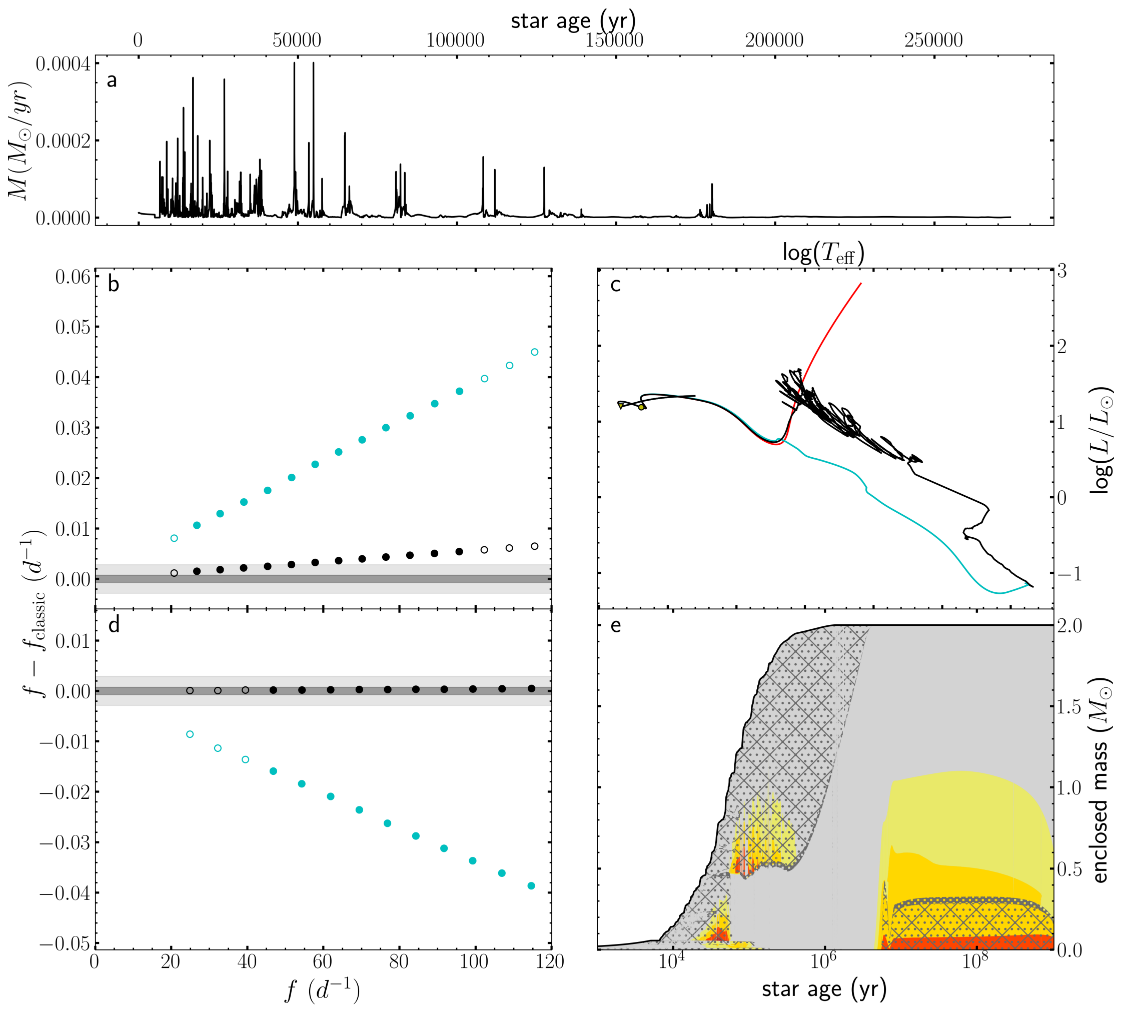

Supplementary Figure 34: Comparison between classical, constant accretion, and disk-mediated accretion model #33 for the standard input physics. **a** The adopted accretion rate as a function of star age. **b** The frequency difference of $l = 1, m = 1$ modes as a function of the pulsation frequency for the predefined pre-main sequence stage. The black (turquoise) circles correspond to differences between the disk-mediated accretion (constant accretion) model and the classical model. Unstable modes are filled, while stable modes are depicted as open circles. The grey areas mark the Rayleigh limit corresponding to 4-year Kepler (dark grey) and 357-days TESS light curves (light grey). **c** The evolution of the classical model (red), the constant accretion model (turquoise) and the disk-mediated accretion model (black) in the Hertzsprung-Russell. **d** Same as panel b but for the evolution model at the ZAMS. **e** Kippenhahn diagram of the evolution (same style as in Figure 1).

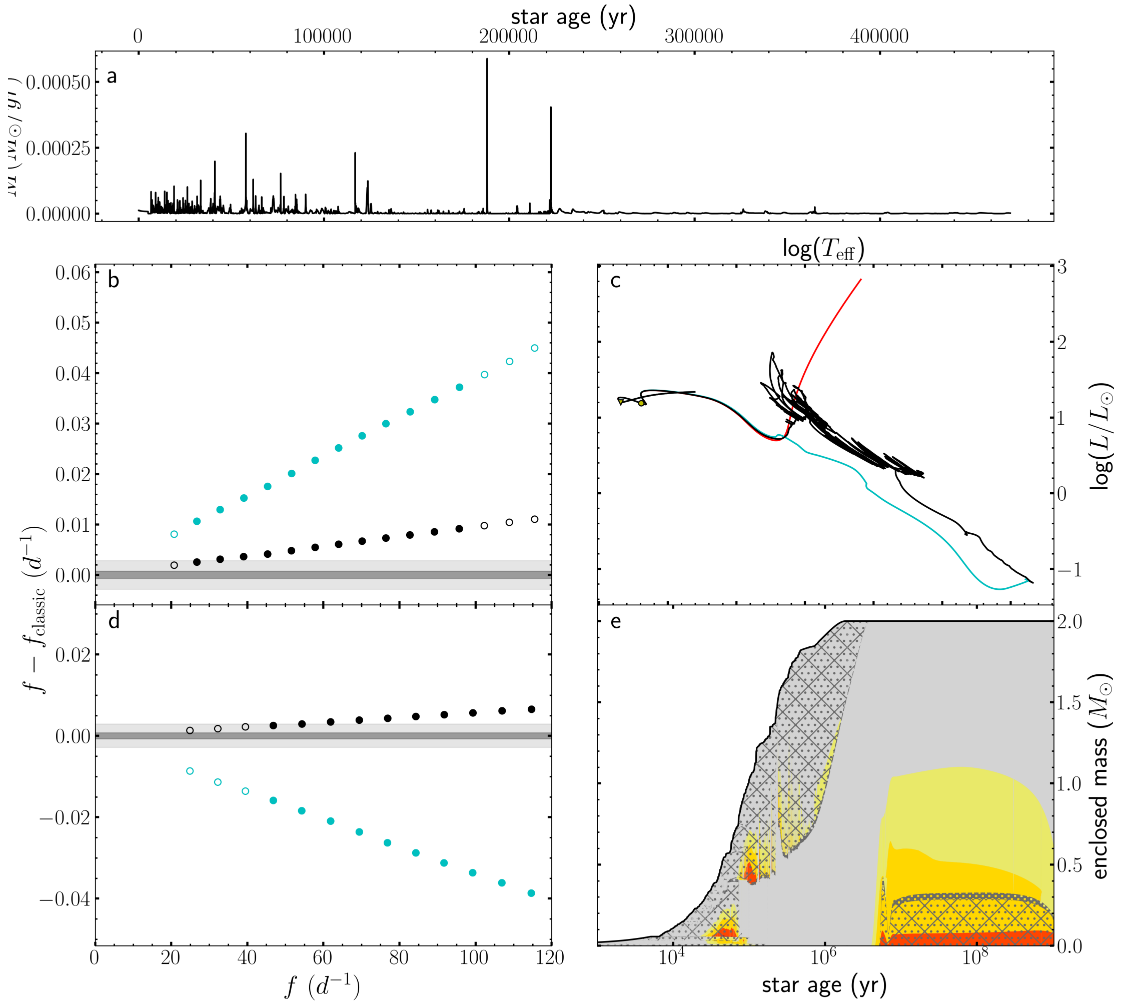

Supplementary Figure 35: Comparison between classical, constant accretion, and disk-mediated accretion model #34 for the standard input physics. **a** The adopted accretion rate as a function of star age. **b** The frequency difference of $l = 1, m = 1$ modes as a function of the pulsation frequency for the predefined pre-main sequence stage. The black (turquoise) circles correspond to differences between the disk-mediated accretion (constant accretion) model and the classical model. Unstable modes are filled, while stable modes are depicted as open circles. The grey areas mark the Rayleigh limit corresponding to 4-year Kepler (dark grey) and 357-days TESS light curves (light grey). **c** The evolution of the classical model (red), the constant accretion model (turquoise) and the disk-mediated accretion model (black) in the Hertzsprung-Russell. **d** Same as panel b but for the evolution model at the ZAMS. **e** Kippenhahn diagram of the evolution (same style as in Figure 1).

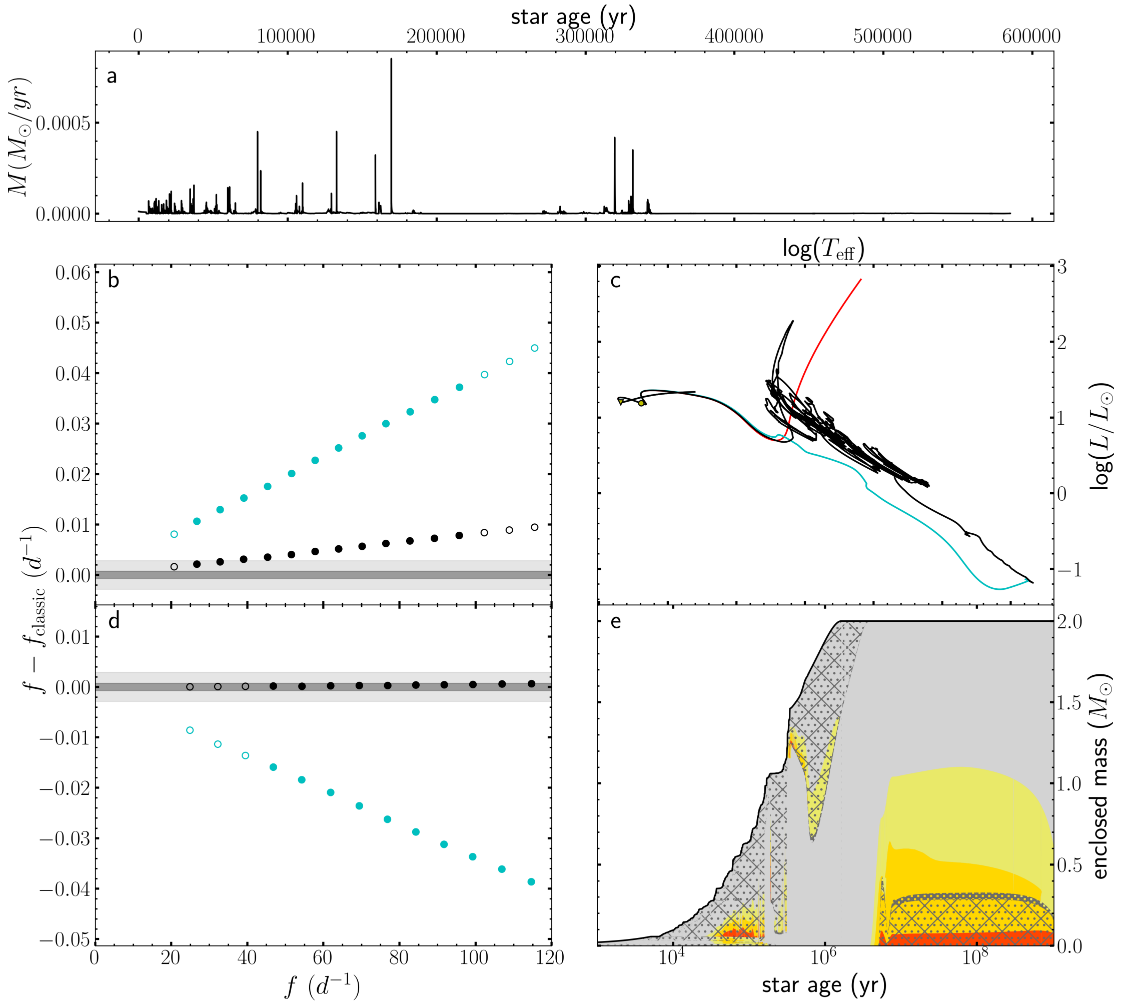

Supplementary Figure 36: Comparison between classical, constant accretion, and disk-mediated accretion model #35 for the standard input physics. a The adopted accretion rate as a function of star age. **b** The frequency difference of $l = 1, m = 1$ modes as a function of the pulsation frequency for the predefined pre-main sequence stage. The black (turquoise) circles correspond to differences between the disk-mediated accretion (constant accretion) model and the classical model. Unstable modes are filled, while stable modes are depicted as open circles. The grey areas mark the Rayleigh limit corresponding to 4-year Kepler (dark grey) and 357-days TESS light curves (light grey). **c** The evolution of the classical model (red), the constant accretion model (turquoise) and the disk-mediated accretion model (black) in the Hertzsprung-Russell. **d** Same as panel b but for the evolution model at the ZAMS. **e** Kippenhahn diagram of the evolution (same style as in Figure 1).

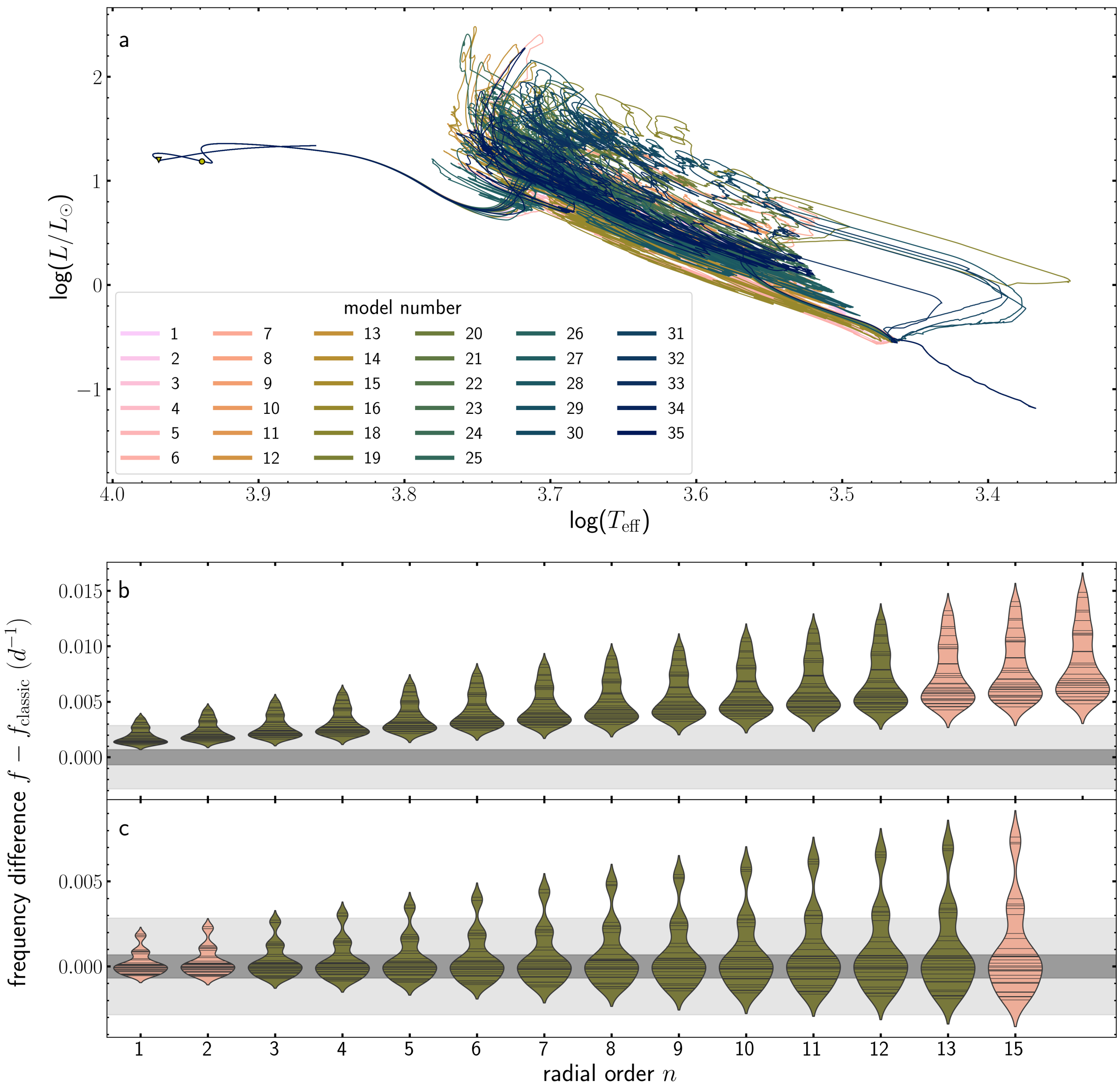

Supplementary Figure 37: Comparison between the different disk-mediated accretion histories for the standard input physics. This figure is the same as Figure 5 but showing the evolutionary tracks of all 34 models. **a** Evolution in a Hertzsprung-Russell diagram. **b** The distribution of frequency values in the 34 models for the pre-main sequence phase. The frequency differences are presented as violin plots grouped according to the radial order. In this type of plot, each horizontal line corresponds to the value of one model and a kernel density estimate calculated with a bandwidth of 0.3 on each side leads to a violin-like shape. Violins are plotted green if the modes are excited in most of the models or salmon if they are stable. The grey areas mark the Rayleigh limit corresponding to 4 year Kepler (dark grey) and 357 days TESS light curves (light grey). The colours

of the evolutionary tracks in the top panel are chosen without specific reasoning and are only to allow discrimination between the models. **c** Distribution of frequency values at the ZAMS.

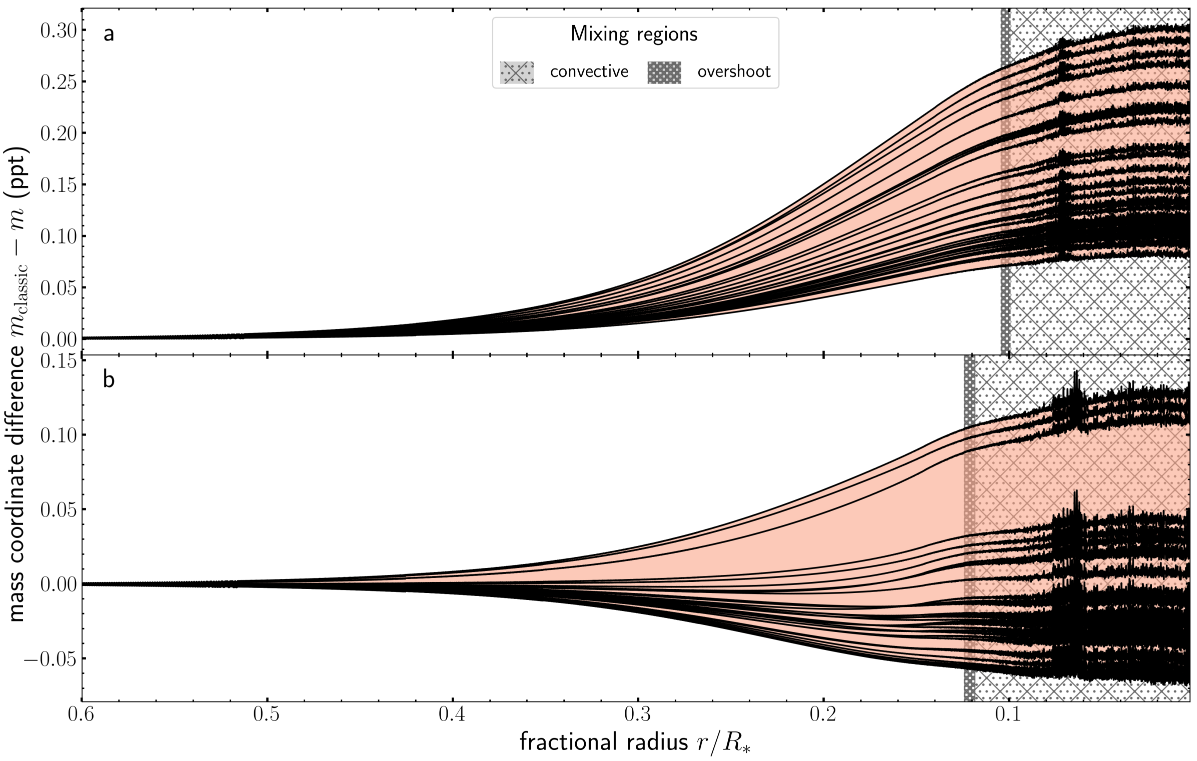

Supplementary Figure 38: Differences in stellar structure between the disk-mediated accretion models and the classical model. This figure is the same as Figure 6 but showing all 34 models. The mass coordinate as a function of the fractional stellar radius. The gray shaded areas mark the extension of the convective core and the overshooting region according to the legend. The salmon shaded area marks the range of mass coordinate differences reached by the 34 disk-mediated models. **a** Differences in structure at the pre-main sequence stage. **b** Differences in structure at the ZAMS.

Supplementary figures - Influence of input physics on the resulting frequency differences

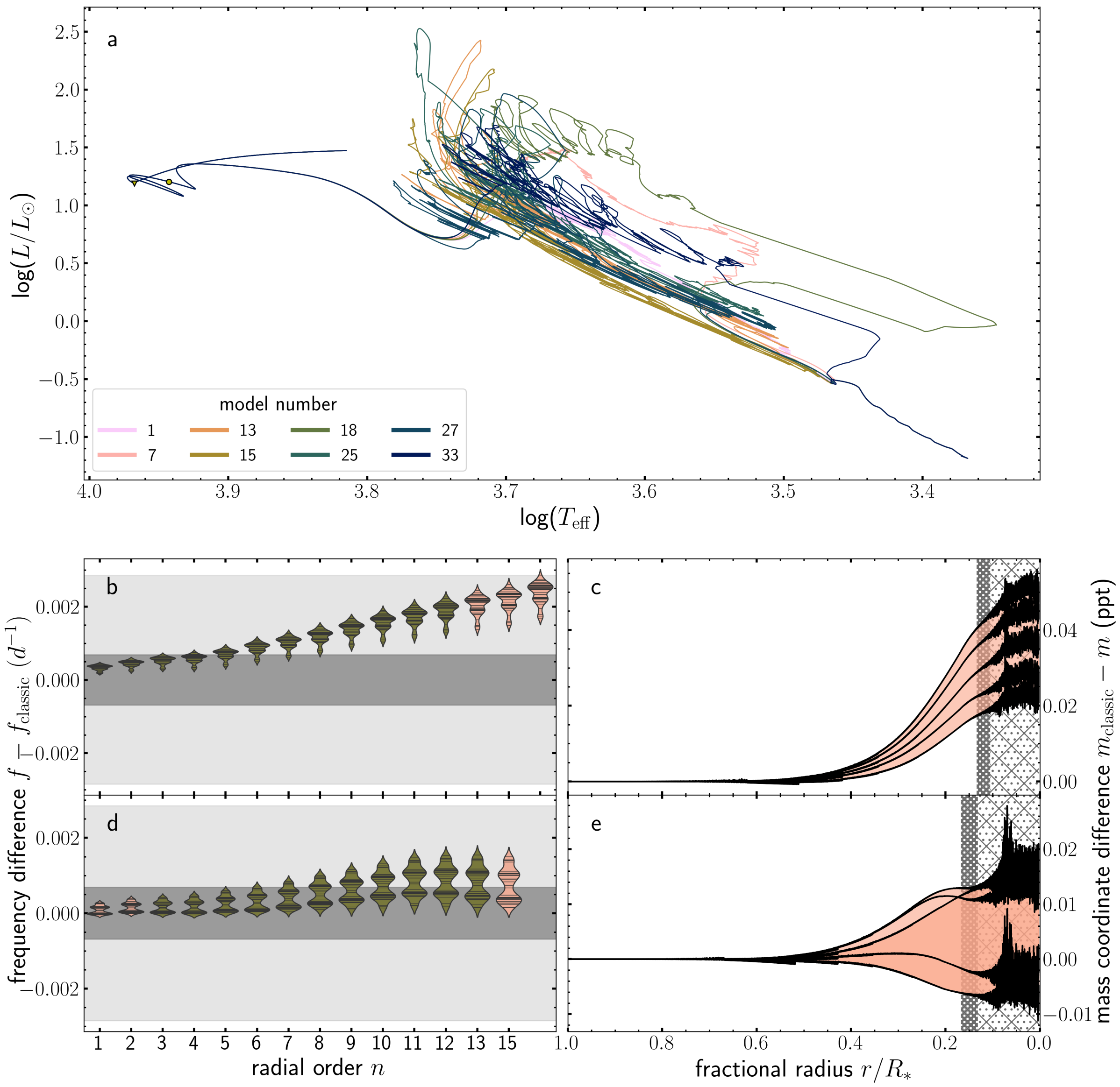

Supplementary Figure 39: Overview of the model results for the input physics with enhanced envelope mixing. For these calculations, the envelope mixing has been set to $5\,\mathrm{cm}^2\,\mathrm{s}^{-1}$ instead of $1\,\mathrm{cm}^2\,\mathrm{s}^{-1}$ used in our standard input physics. **a** The evolution of eight chosen disk-mediated models in the Hertzsprung Russell diagram. See Supplementary Figure 40 for a version with all models shown. **b** Frequency differences at the pre-main sequence stage as violin plots for all models. Equivalent to panel a of Figure 7. **c** The differences in internal structure: mass coordinate as a function fractional radius at the pre-main sequence stage. Similar to Figure 6. Models shown are from top to bottom:

31, 15, and 6. **d** Same as panel b but for the evolution models at the ZAMS. **e** Same as panel c but for the evolution models at the ZAMS. Models shown are from top to bottom: 16, 31, 13, 5, and 8.

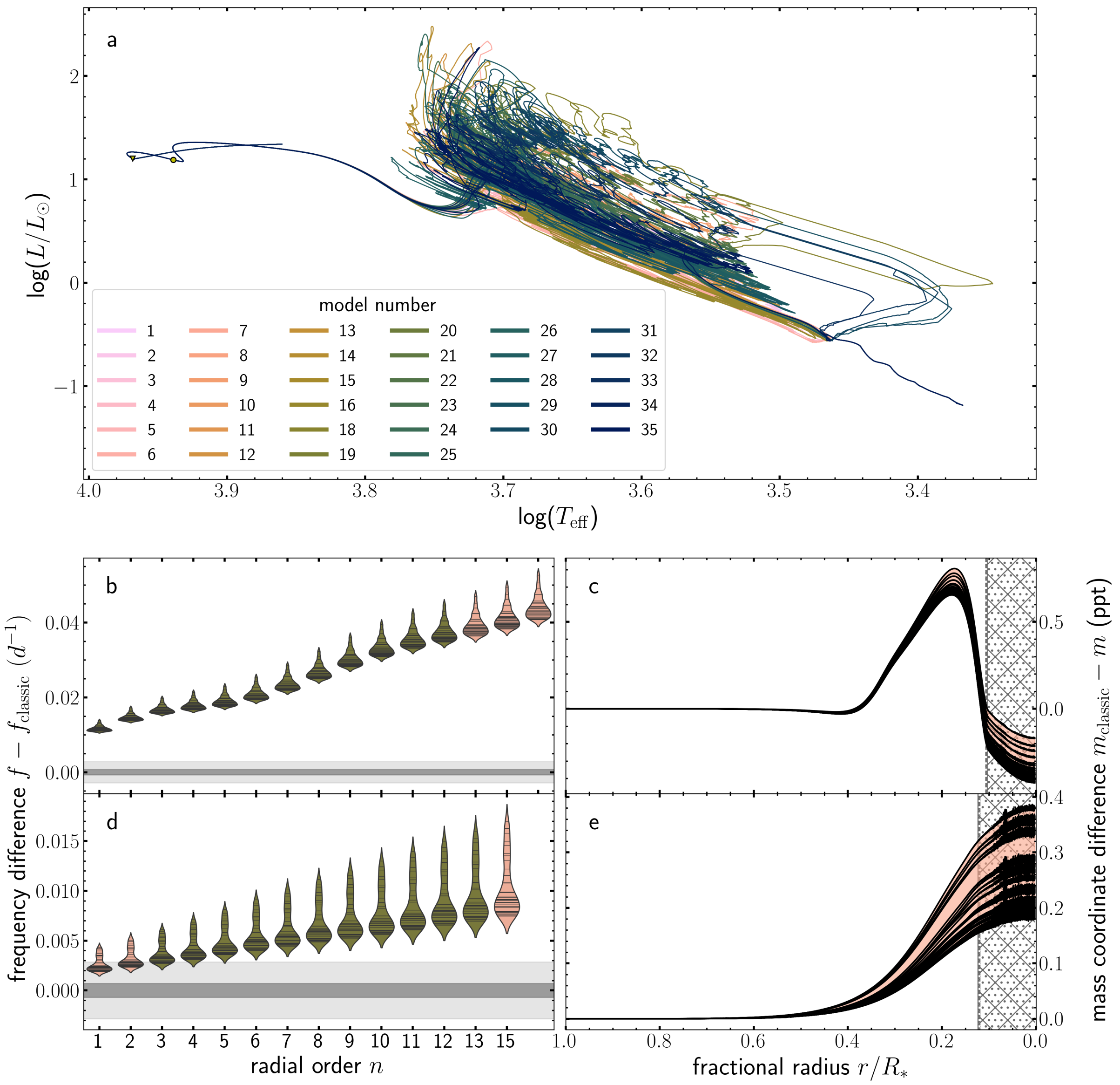

Supplementary Figure 40: Overview of the model results for the input physics with enhanced envelope mixing. This figure is the same as Supplementary Figure 39 but showing all 34 models in all panels. For these calculations, the envelope mixing has been set to $5\,\mathrm{cm}^2\,\mathrm{s}^{-1}$ instead of $1\,\mathrm{cm}^2\,\mathrm{s}^{-1}$ used in our standard input physics. **a** The evolution of all disk-mediated models in the Hertzsprung Russell diagram. **b** Frequency differences at the pre-main sequence stage as violin plots. Equivalent to panel a of Figure 7. **c** The differences in internal structure: mass coordinate as a function fractional radius at the pre-main sequence stage. Similar to Figure 6. **d** Same as panel b but for the evolution models at the ZAMS. **e** Same as panel c but for the evolution models at the ZAMS.

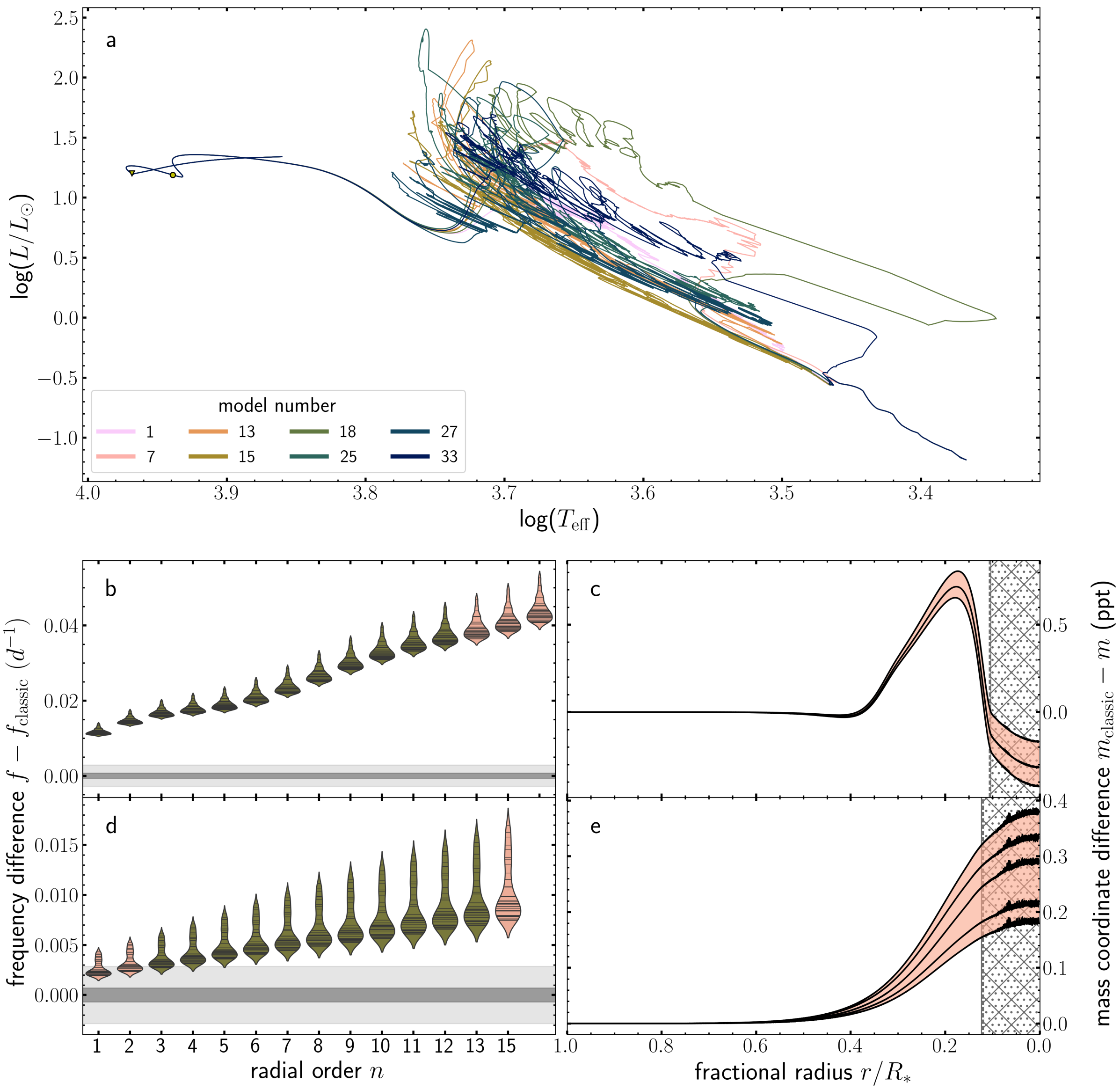

Supplementary Figure 41: Overview of the model results for the input physics with enhanced overshooting. For these calculations, the overshooting coefficients have been set to $f = 0.05$ and $f_0 = 0.025$ at the top and $f = 0.025$ and $f_0 = 0.0125$ at the bottom, which is five times the value for the standard input physics used in our standard input physics. **a** The evolution of eight chosen disk-mediated models in the Hertzsprung Russell diagram. **b** Frequency differences at the pre-main sequence stage as violin plots. Equivalent to panel a of Figure 7. **c** The differences in internal structure: mass coordinate as a function fractional radius at the pre-main sequence stage. Similar to Figure 6. Models shown are from top to bottom: 5, 24, 29, 26 and 6.**d** Same as panel b but for the evolution models at the ZAMS. **e** Same as panel c but for the evolution models at the ZAMS. Models

shown are from top to bottom: 11, 27, 6, 32, and 22.

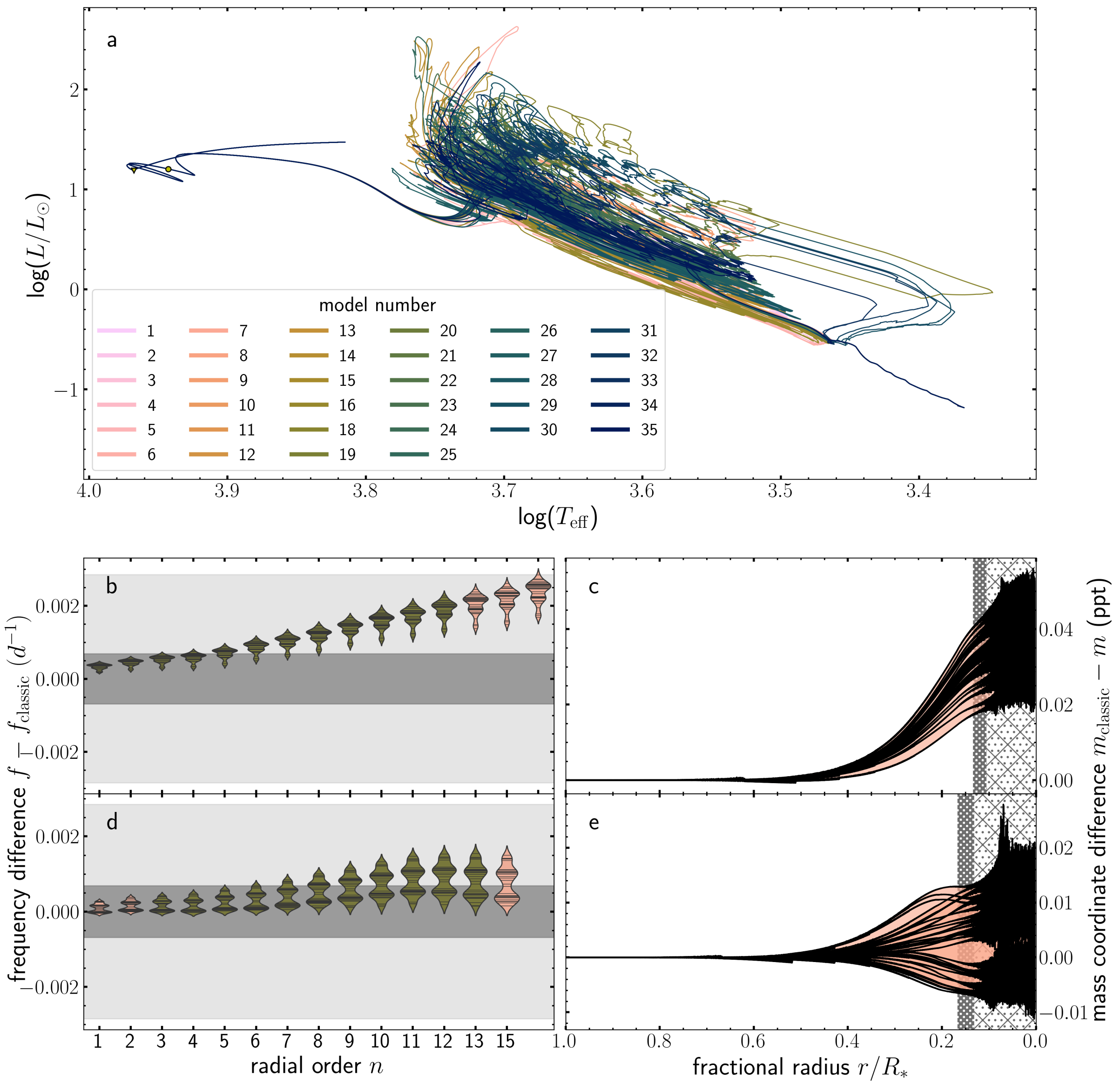

Supplementary Figure 42: Overview of the model results for the input physics with enhanced overshooting. This figure is the same as Supplementary Figure 41 but showing all 34 models in all panels. For these calculations, the overshooting coefficients have been set to $f = 0.05$ and $f_0 = 0.025$ at the top and $f = 0.025$ and $f_0 = 0.0125$ at the bottom, which is five times the value for the standard input physics used in our standard input physics. **a** The evolution of all disk-mediated models in the Hertzsprung Russell diagram. **b** Frequency differences at the pre-main sequence stage as violin plots. Equivalent to panel a of Figure 7. **c** The differences in internal structure: mass coordinate as a function fractional radius at the pre-main sequence stage. Similar to Figure 6. **d** Same as panel b but for the evolution models at the ZAMS. **e** Same as panel c but for

the evolution models at the ZAMS.

Supplementary figure - Results for the frequency differences at an earlier evolutionary phase

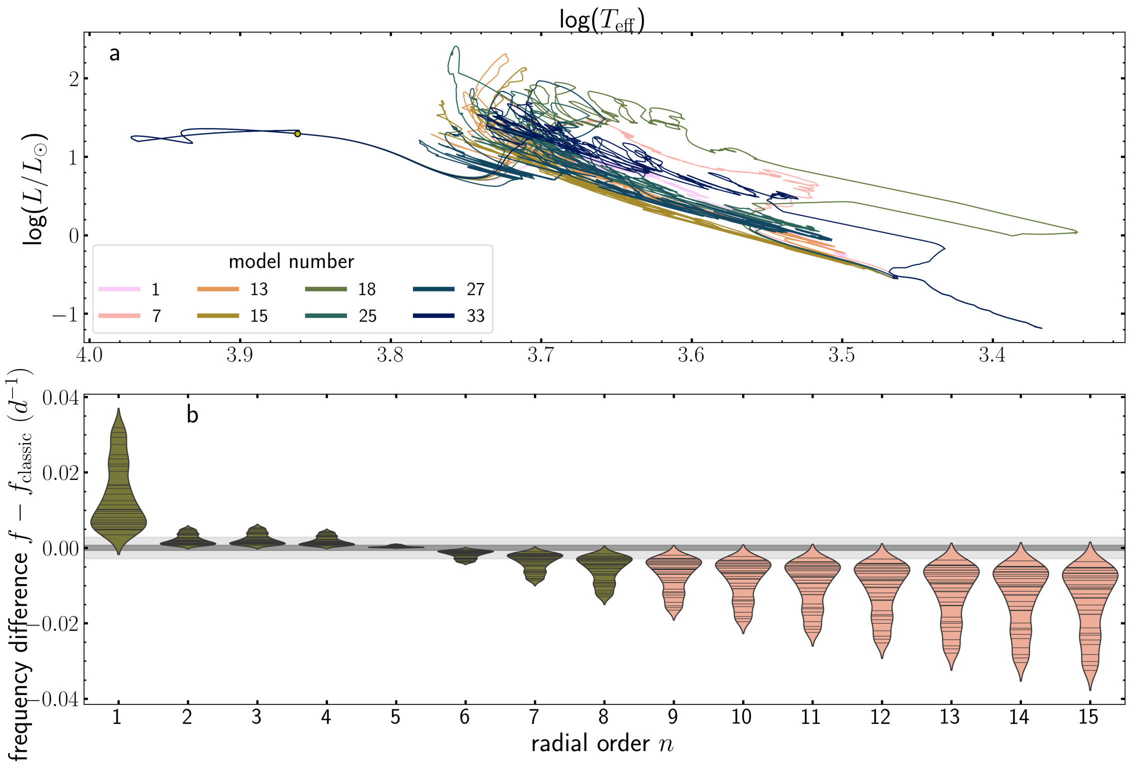

Supplementary Figure 43: Overview of the model results for the standard input physics at an earlier evolutionary stage as presented in the main text. The results here are presented at the moment in the evolution at which the stellar radius reaches 2.8 $R_\odot$ (see methods, subsection stellar evolution models). **a** Eight chosen evolutionary tracks in the Hertzsprung-Russell diagram. The yellow circle marks the evolutionary stage of 2.8 $R_\odot$. **b** Frequency differences as violin plots.

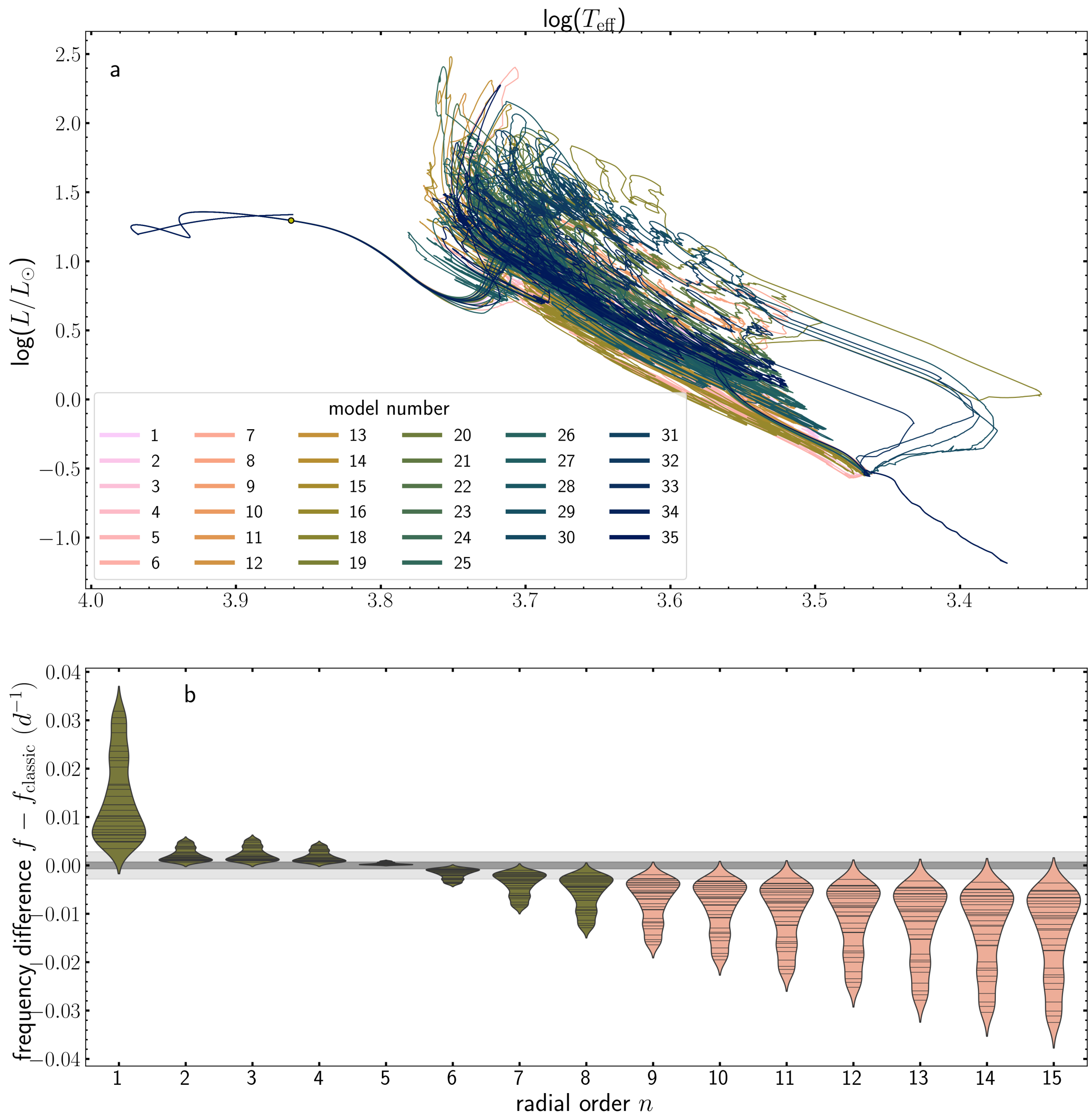

Supplementary Figure 44: Overview of the model results for the standard input physics at an earlier evolutionary stage as presented in the main text. This figure is the same as Supplementary Figure 43 but showing all 34 models in all panels. The results here are presented at the moment in the evolution at which the stellar radius reaches 2.8 $R_\odot$ (see methods, subsection stellar evolution models). **a** All evolutionary tracks in the Hertzsprung-Russell diagram. The yellow circle marks the evolutionary stage of 2.8 $R_\odot$. **b** Frequency differences as violin plots.
